# Supplementary material for: A cross-disease, pleiotropy-driven approach for therapeutic target prioritization and evaluation
Source: Cell Rep Methods. 2024 Apr 16;4(4):100757. doi: 10.1016/j.crmeth.2024.100757 (PMC11046034; doi:10.1016/j.crmeth.2024.100757)
Supplement: Document S1. Figures S1–S6 [file mmc1.pdf]

Cell Reports Methods, Volume *4*

Supplemental information

A cross-disease, pleiotropy-driven approach for therapeutic target prioritization and evaluation

Chaohui Bao, Tingting Tan, Shan Wang, Chenxu Gao, Chang Lu, Siyue Yang, Yizhu Diao, Lulu Jiang, Duohui Jing, Liye Chen, Haitao Lv, and Hai Fang

Supplemental Figures

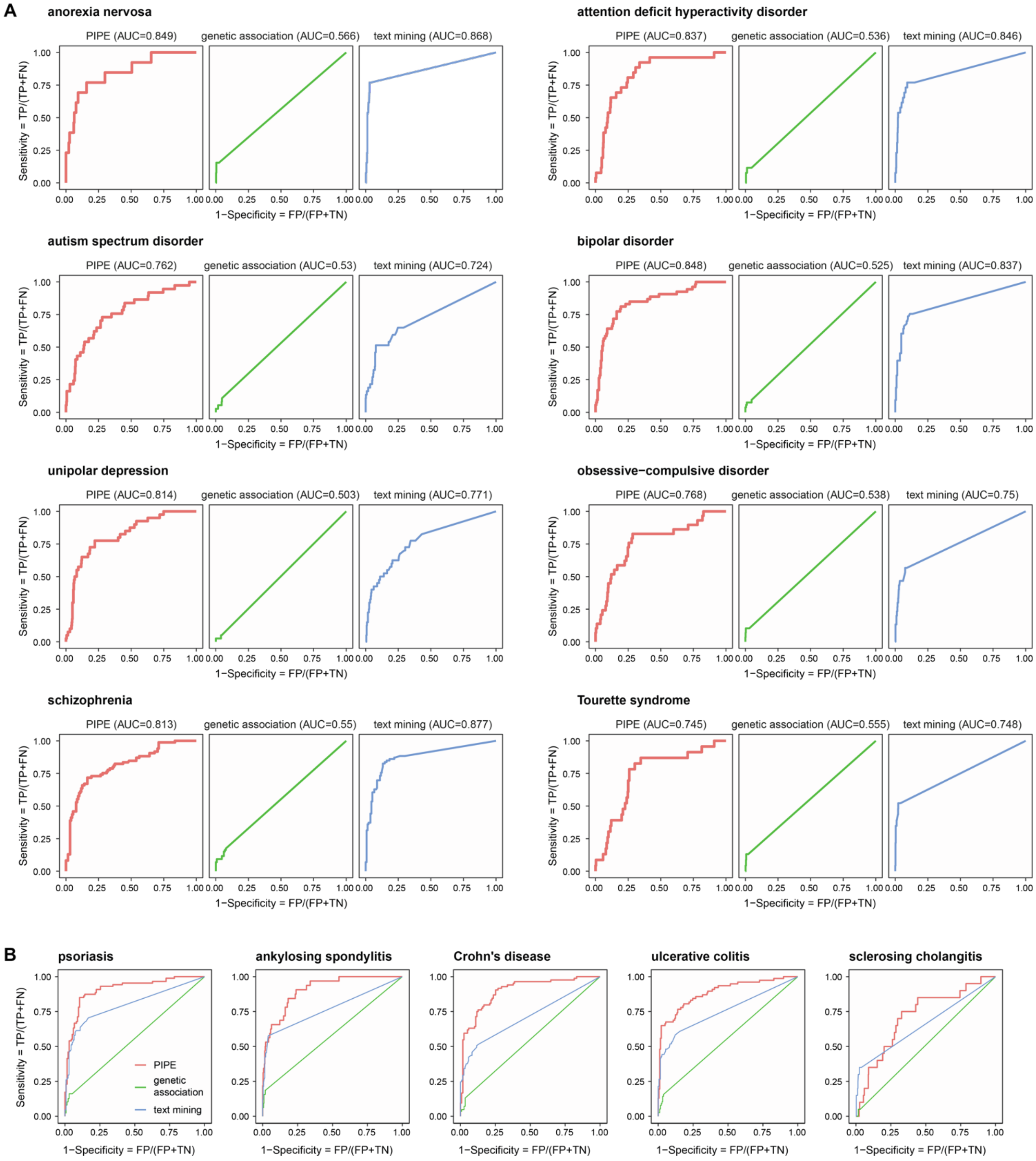

Figure S1. AUC plots comparing the performance between PIPE and Open Targets (including ‘genetic association’ and ‘text mining’), related to Figure 2A and Figure 4A. FN, false negatives; FP, false positives; TN, true negatives; TP: true positives. **(A)** AUC plots for each individual disease within neuropsychiatric disorders. **(B)** AUC plots for each individual disease within inflammatory disorders.

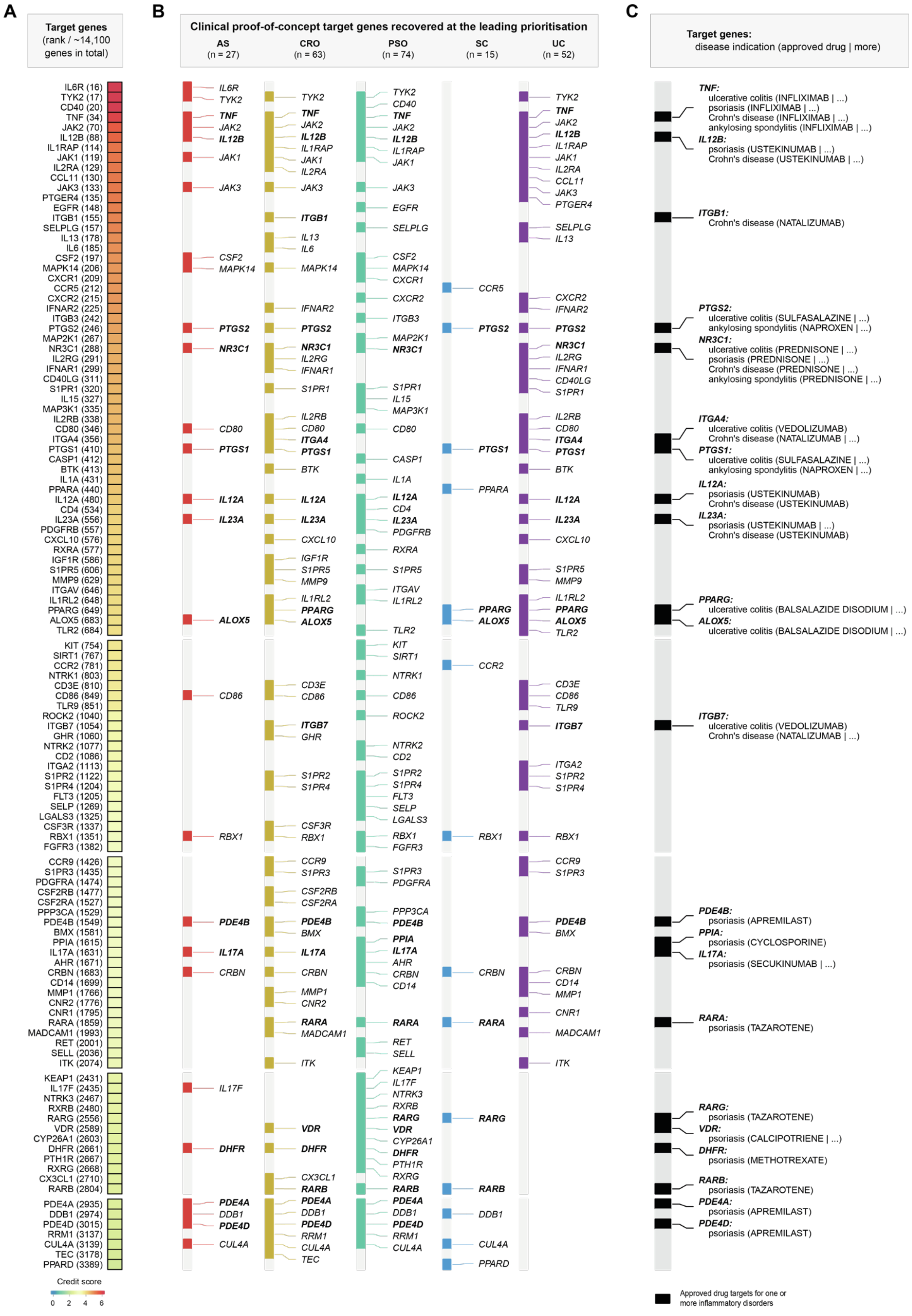
A
Target genes
(rank / ~14,100 genes in total)
IL6R (16)
TYK2 (17)
CD40 (20)
TNF (34)
JAK2 (70)
IL12B (88)
IL1RAP (114)
JAK1 (119)
IL2RA (129)
CCL11 (130)
JAK3 (133)
PTGER4 (135)
EGFR (148)
ITGB1 (155)
SELPLG (157)
IL13 (178)
IL6 (185)
CSF2 (197)
MAPK14 (206)
CXCR1 (209)
CCR5 (212)
CXCR2 (215)
IFNAR2 (225)
ITGB3 (242)
PTGS2 (246)
MAP2K1 (267)
NR3C1 (288)
IL2RG (291)
IFNAR1 (299)
CD40LG (311)
S1PR1 (320)
IL15 (327)
MAP3K1 (335)
IL2RB (338)
CD80 (346)
ITGA4 (356)
PTGS1 (410)
CASP1 (412)
BTK (413)
IL1A (431)
PPARA (440)
IL12A (480)
CD4 (534)
IL23A (556)
PDGFRB (557)
CXCL10 (576)
RXRA (577)
IGF1R (586)
S1PR5 (606)
MMP9 (629)
ITGAV (646)
IL1RL2 (648)
PPARG (649)
ALOX5 (683)
TLR2 (684)
KIT (754)
SIRT1 (767)
CCR2 (781)
NTRK1 (803)
CD3E (810)
CD86 (849)
TLR9 (851)
ROCK2 (1040)
ITGB7 (1054)
GHR (1060)
NTRK2 (1077)
CD2 (1086)
ITGA2 (1113)
S1PR2 (1122)
S1PR4 (1204)
FLT3 (1205)
SELP (1269)
LGALS3 (1325)
CSF3R (1337)
RBX1 (1351)
FGFR3 (1382)
CCR9 (1426)
S1PR3 (1435)
PDGFRA (1474)
CSF2RB (1477)
CSF2RA (1527)
PPP3CA (1529)
PDE4B (1549)
BMX (1581)
PPIA (1615)
IL17A (1631)
AHR (1671)
CRBN (1683)
CD14 (1699)
MMP1 (1766)
CNR2 (1776)
CNR1 (1795)
RARA (1859)
MADCAM1 (1993)
RET (2001)
SELL (2036)
ITK (2074)
KEAP1 (2431)
IL17F (2435)
NTRK3 (2467)
RXRB (2480)
RARG (2556)
VDR (2589)
CYP26A1 (2603)
DHFR (2661)
PTH1R (2667)
RXRG (2668)
CX3CL1 (2710)
RARB (2804)
PDE4A (2935)
DDB1 (2974)
PDE4D (3015)
RRM1 (3137)
CUL4A (3139)
TEC (3178)
PPARD (3389)
Credit score
0 2 4 6
B
Clinical proof-of-concept target genes recovered at the leading prioritisation
AS (n = 27)
IL6R, TYK2, TNF, JAK2, IL12B, JAK1, JAK3, CSF2, MAPK14, PTGS2, NR3C1, CD80, PTGS1, IL12A, IL23A, ALOX5, CD86, RBX1, PDE4B, IL17A, CRBN, IL17F, DHFR, PDE4A, DDB1, PDE4D, CUL4A
CRO (n = 63)
TYK2, TNF, JAK2, IL12B, IL1RAP, JAK1, IL2RA, JAK3, ITGB1, IL13, IL6, MAPK14, IFNAR2, PTGS2, NR3C1, IL2RG, IFNAR1, S1PR1, IL2RB, CD80, ITGA4, PTGS1, BTK, IL12A, IL23A, CXCL10, IGF1R, S1PR5, MMP9, IL1RL2, PPARG, ALOX5, CD3E, CD86, ITGB7, GHR, S1PR2, S1PR4, CSF3R, RBX1, CCR9, S1PR3, CSF2RB, CSF2RA, PDE4B, BMX, IL17A, CRBN, MMP1, CNR2, RARA, MADCAM1, ITK, VDR, DHFR, CX3CL1, RARB, PDE4A, DDB1, PDE4D, RRM1, CUL4A, TEC
PSO (n = 74)
TYK2, CD40, TNF, JAK2, IL12B, IL1RAP, JAK1, JAK3, EGFR, SELPLG, CSF2, MAPK14, CXCR1, CXCR2, ITGB3, MAP2K1, NR3C1, S1PR1, IL15, MAP3K1, CD80, CASP1, IL1A, IL12A, CD4, IL23A, PDGFRB, RXRA, S1PR5, ITGAV, IL1RL2, TLR2, KIT, SIRT1, NTRK1, CD86, ROCK2, NTRK2, CD2, S1PR2, S1PR4, FLT3, SELP, LGALS3, RBX1, FGFR3, S1PR3, PDGFRA, PPP3CA, PDE4B, PPIA, IL17A, AHR, CRBN, CD14, RARA, RET, SELL, KEAP1, IL17F, NTRK3, RXRB, RARG, VDR, CYP26A1, DHFR, PTH1R, RXRG, RARB, PDE4A, DDB1, PDE4D, RRM1, CUL4A
SC (n = 15)
CCR5, PTGS2, PTGS1, PPARA, PPARG, ALOX5, CCR2, RBX1, CRBN, RARA, RARG, RARB, DDB1, CUL4A, PPARD
UC (n = 52)
TYK2, TNF, JAK2, IL12B, IL1RAP, JAK1, IL2RA, CCL11, JAK3, PTGER4, SELPLG, IL13, CXCR2, IFNAR2, PTGS2, NR3C1, IL2RG, IFNAR1, CD40LG, S1PR1, IL2RB, CD80, ITGA4, PTGS1, BTK, IL12A, IL23A, CXCL10, S1PR5, MMP9, IL1RL2, PPARG, ALOX5, TLR2, CD3E, CD86, TLR9, ITGB7, ITGA2, S1PR2, S1PR4, RBX1, CCR9, S1PR3, PDE4B, BMX, CRBN, CD14, MMP1, CNR1, MADCAM1, ITK
C
Target genes:
disease indication (approved drug | more)
TNF:
ulcerative colitis (INFLIXIMAB | ...)
psoriasis (INFLIXIMAB | ...)
Crohn's disease (INFLIXIMAB | ...)
ankylosing spondylitis (INFLIXIMAB | ...)
IL12B:
psoriasis (USTEKINUMAB | ...)
Crohn's disease (USTEKINUMAB | ...)
ITGB1:
Crohn's disease (NATALIZUMAB)
PTGS2:
ulcerative colitis (SULFASALAZINE | ...)
ankylosing spondylitis (NAPROXEN | ...)
NR3C1:
ulcerative colitis (PREDNISONE | ...)
psoriasis (PREDNISONE | ...)
Crohn's disease (PREDNISONE | ...)
ankylosing spondylitis (PREDNISONE | ...)
ITGA4:
ulcerative colitis (VEDOLIZUMAB)
Crohn's disease (NATALIZUMAB | ...)
PTGS1:
ulcerative colitis (SULFASALAZINE | ...)
ankylosing spondylitis (NAPROXEN | ...)
IL12A:
psoriasis (USTEKINUMAB)
Crohn's disease (USTEKINUMAB)
IL23A:
psoriasis (USTEKINUMAB | ...)
Crohn's disease (USTEKINUMAB)
PPARG:
ulcerative colitis (BALSALAZIDE DISODIUM | ...)
ALOX5:
ulcerative colitis (BALSALAZIDE DISODIUM | ...)
ITGB7:
ulcerative colitis (VEDOLIZUMAB)
Crohn's disease (NATALIZUMAB | ...)
PDE4B:
psoriasis (APREMILAST)
PPIA:
psoriasis (CYCLOSPORINE)
IL17A:
psoriasis (SECUKINUMAB | ...)
RARA:
psoriasis (TAZAROTENE)
RARG:
psoriasis (TAZAROTENE)
VDR:
psoriasis (CALCIPOTRIENE | ...)
DHFR:
psoriasis (METHOTREXATE)
RARB:
psoriasis (TAZAROTENE)
PDE4A:
psoriasis (APREMILAST)
PDE4D:
psoriasis (APREMILAST)
Approved drug targets for one or more inflammatory disorders

Figure S2. Illustration of clinical proof-of-concept therapeutic targets recovered at the leading prioritisation for each individual disease within inflammatory disorders, related to Figure 4. (A) The heatmap for clinical proof-of-concept therapeutic targets color-coded by the credit score. The number in the parentheses indicates the target rank. **(B)** Annotations to clinical proof-of-concept targets per disease as indicated. **(C)** Annotations for approved drug targets, disease indications, and approved drugs. This illustration implicates drug repurposing opportunities within inflammatory disorders.

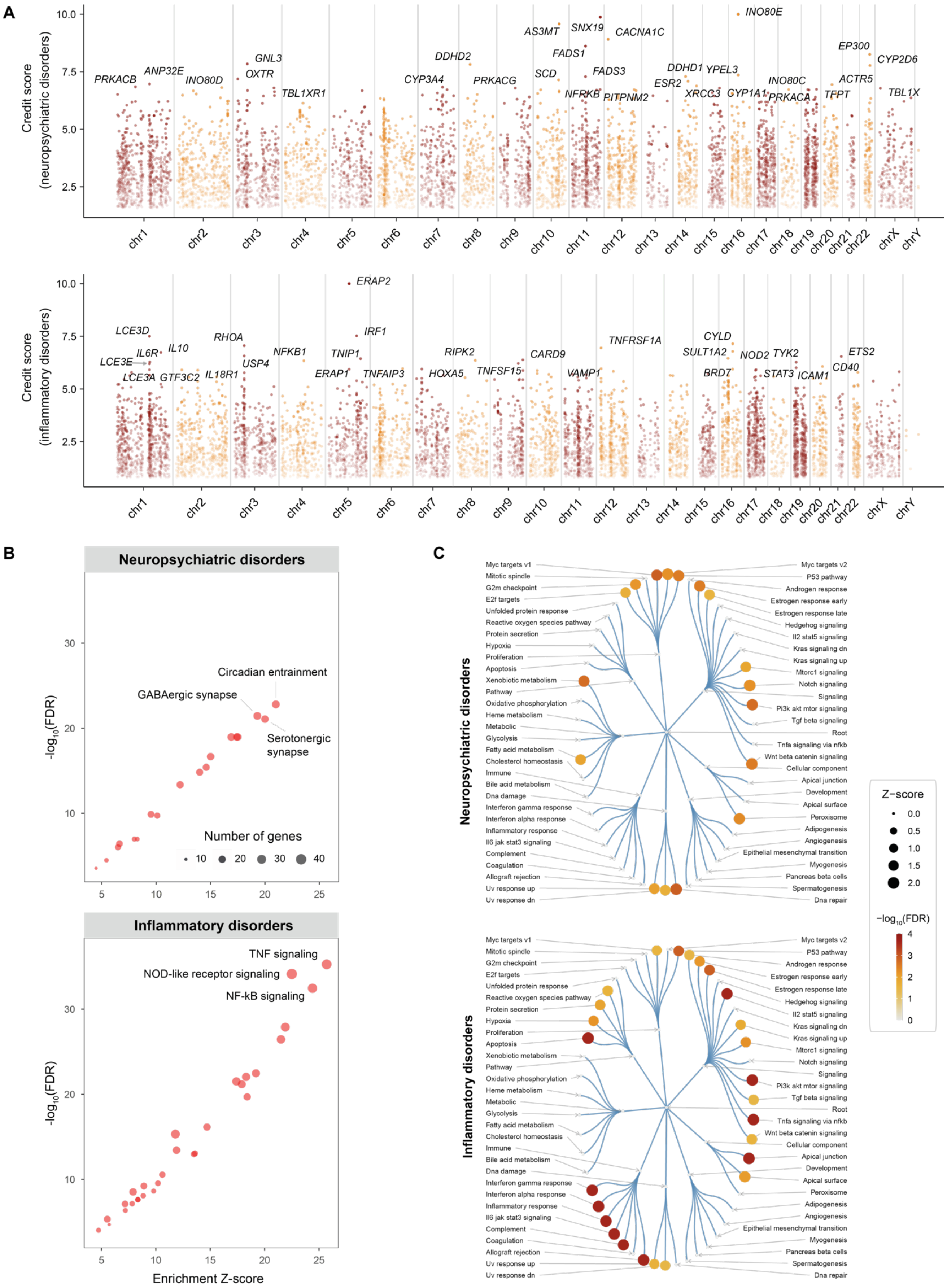

A
Credit score (neuropsychiatric disorders)
10.0
7.5
5.0
2.5
PRKACB
ANP32E
INO80D
GNL3
OXTR
TBL1XR1
DDHD2
CYP3A4
PRKACG
AS3MT
SNX19
CACNA1C
FADS1
SCD
FADS3
NFRKB
PITPNM2
DDHD1
YPEL3
ESR2
XRCC3
CYP1A1
INO80E
INO80C
PRKACA
EP300
CYP2D6
ACTR5
TFPT
TBL1X
chr1
chr2
chr3
chr4
chr5
chr6
chr7
chr8
chr9
chr10
chr11
chr12
chr13
chr14
chr15
chr16
chr17
chr18
chr19
chr20
chr21
chr22
chrX
chrY
Credit score (inflammatory disorders)
ERAP2
LCE3D
IL6R
IL10
LCE3E
LCE3A
GTF3C2
RHOA
USP4
IL18R1
NFKB1
TNIP1
IRF1
ERAP1
TNFAIP3
RIPK2
HOXA5
TNFSF15
CARD9
VAMP1
TNFRSF1A
CYLD
SULT1A2
BRD7
NOD2
TYK2
STAT3
ICAM1
ETS2
CD40
B
Neuropsychiatric disorders
-log10(FDR)
30
20
10
Circadian entrainment
GABAergic synapse
Serotonergic synapse
Number of genes
10
20
30
40
5
10
15
20
25
Inflammatory disorders
TNF signaling
NOD-like receptor signaling
NF-kB signaling
Enrichment Z-score
C
Neuropsychiatric disorders
Inflammatory disorders
Myc targets v1
Mitotic spindle
G2m checkpoint
E2f targets
Unfolded protein response
Reactive oxygen species pathway
Protein secretion
Hypoxia
Proliferation
Apoptosis
Xenobiotic metabolism
Pathway
Oxidative phosphorylation
Heme metabolism
Metabolic
Glycolysis
Fatty acid metabolism
Cholesterol homeostasis
Immune
Bile acid metabolism
Dna damage
Interferon gamma response
Interferon alpha response
Inflammatory response
Il6 jak stat3 signaling
Complement
Coagulation
Allograft rejection
Uv response up
Uv response dn
Myc targets v2
P53 pathway
Androgen response
Estrogen response early
Estrogen response late
Hedgehog signaling
Il2 stat5 signaling
Kras signaling dn
Kras signaling up
Mtorc1 signaling
Notch signaling
Signaling
Pi3k akt mtor signaling
Tgf beta signaling
Root
Tnfa signaling via nfkb
Wnt beta catenin signaling
Cellular component
Apical junction
Development
Apical surface
Peroxisome
Adipogenesis
Angiogenesis
Epithelial mesenchymal transition
Myogenesis
Pancreas beta cells
Spermatogenesis
Dna repair
Z-score
0.0
0.5
1.0
1.5
2.0
-log10(FDR)
4
3
2
1
0

Figure S3. Target genes prioritisations, pathway enrichments, and hallmark enrichments, related to Figure 5. (A) Manhattan plots illustrating credit scores (y-axis) for all prioritised target genes across chromosomes (x-axis), with top 30 named. *Top panel:* neuropsychiatric disorders. *Bottom panel:* inflammatory disorders. **(B)** Dot plots illustrating top KEGG pathways enriched for each system of disorders, with the top 3 named. *Left panel:* neuropsychiatric disorders. *Right panel:* inflammatory disorders. **(C)** Circular illustration of molecular hallmark enrichments for each system of disorders. *Left panel:* neuropsychiatric disorders. *Right panel:* inflammatory disorders.

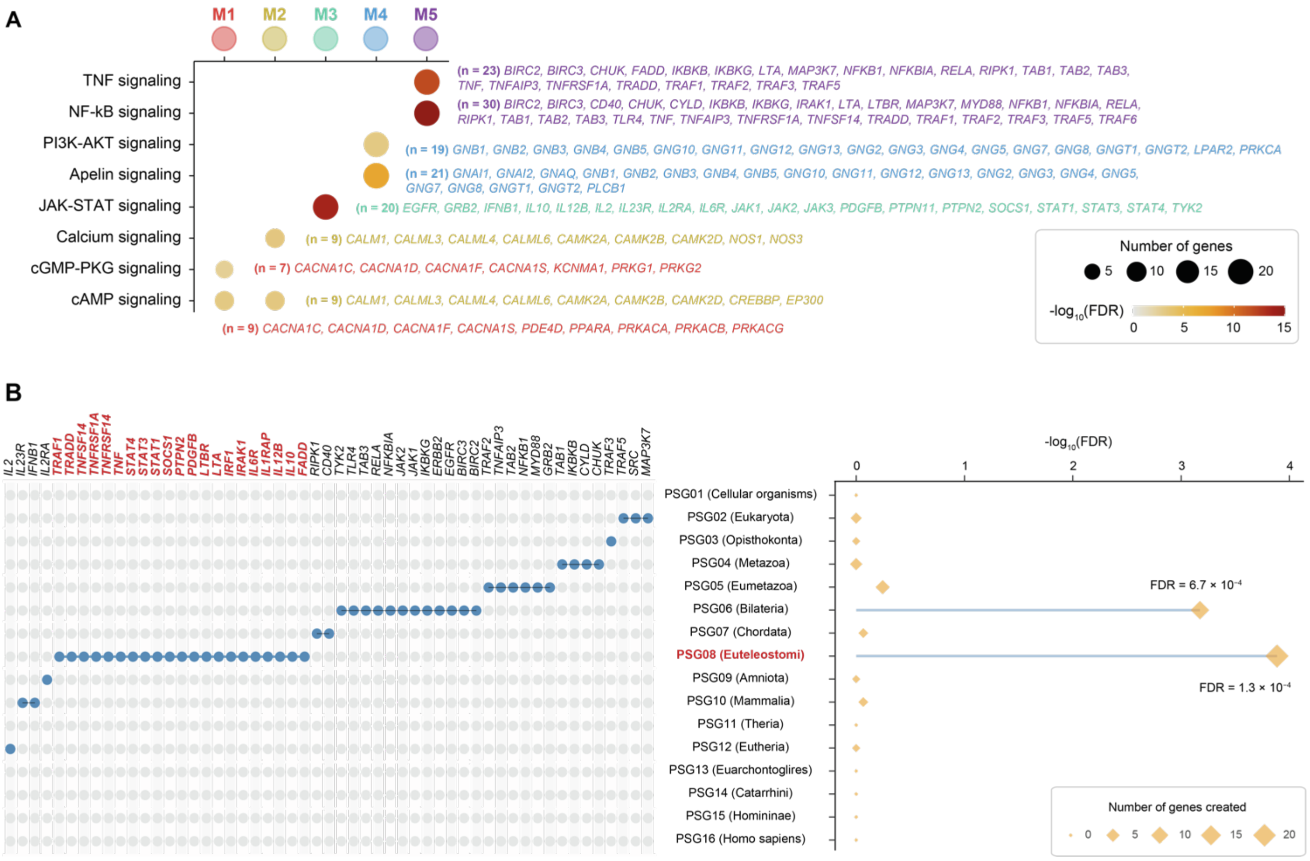

Figure S4. Crosstalk-based network modular analysis, related to Figure 6. (A) KEGG pathway enrichment analysis for genes within each of five modules (M1 – M5). Enrichment analysis was based on one-sided Fisher's exact test. **(B)** Enrichment analysis using phylostrata reveals evolutionary origins of genes in modules specific to inflammatory modules. Kite plot of enriched phylostrata (right panel), with created genes per phylostratum as indicated in blue dots (left panel). Dated through genomic phylostratigraphy (PSG), phylostrata are coded from the earliest (PSG01) to the most recent (PSG16). A phylostratum contains a group of genes that were first created at this phylostratum; for example, genes in red were first created at Euteleostomi, mostly involved in NF-kB signaling (*IRAK1*, *LTA*, *LTBR*, *TNF*, *TNFRSF1A*, *TNFSF14*, *TRADD*, and *TRAF1*) and JAK-STAT signaling (*IL10*, *IL12B*, *IL6R*, *PDGFB*, *PTPN2*, *SOCS1*, *STAT1*, *STAT3*, and *STAT4*).

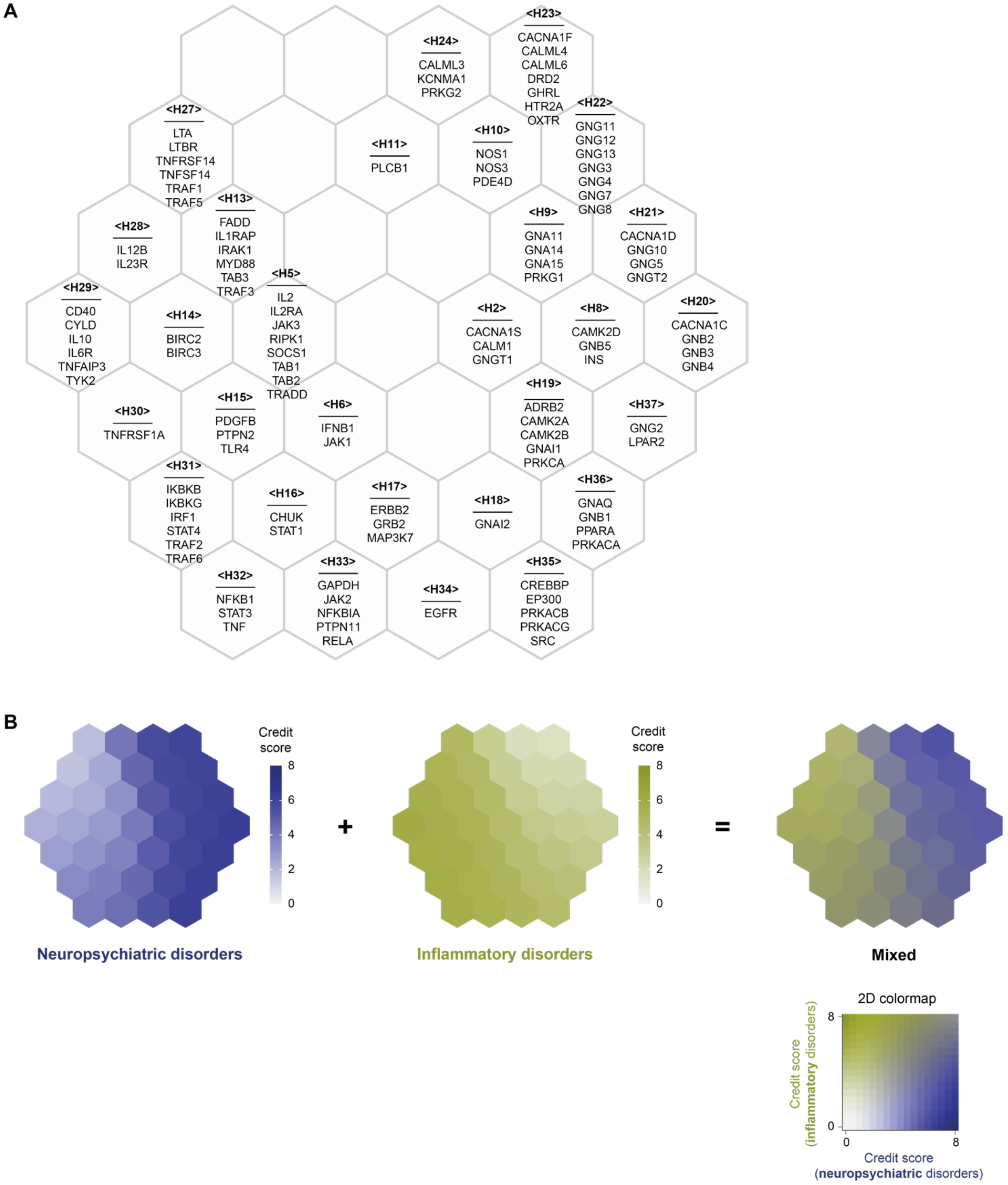

Figure S5. Crosstalk-based prioritisation map analysis, related to Figure 7. (A) A 2D map with target genes listed per hexagon (H1 - H37). **(B)** Prioritisation map coded using two mixed colors.

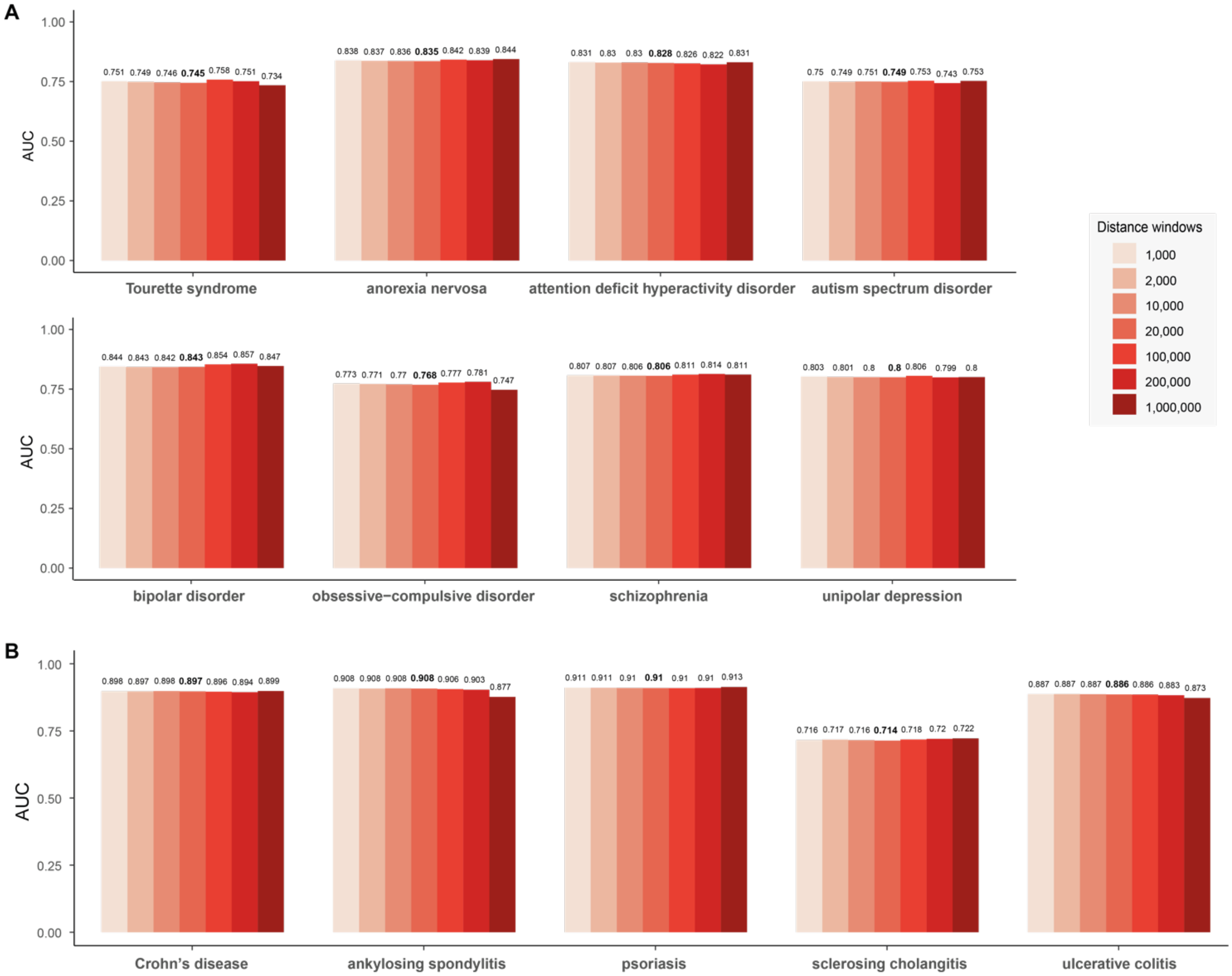

Figure S6. Performance evaluation by changing the distance windows, related to the STAR Methods. (A) Evaluation for neuropsychiatric disorders. **(B)** Evaluation for inflammatory disorders. The texts in bold highlight the selection of a 20kb distance.
