# Supplementary material for: A cross-disease, pleiotropy-driven approach for therapeutic target prioritization and evaluation
Source: Cell Rep Methods. 2024 Apr 16;4(4):100757. doi: 10.1016/j.crmeth.2024.100757 (PMC11046034; doi:10.1016/j.crmeth.2024.100757)
Supplement: Document S2. Article plus supplemental information [file mmc8.pdf]

Cell Reports
Methods

Article

A cross-disease, pleiotropy-driven approach for therapeutic target prioritization and evaluation

Graphical abstract

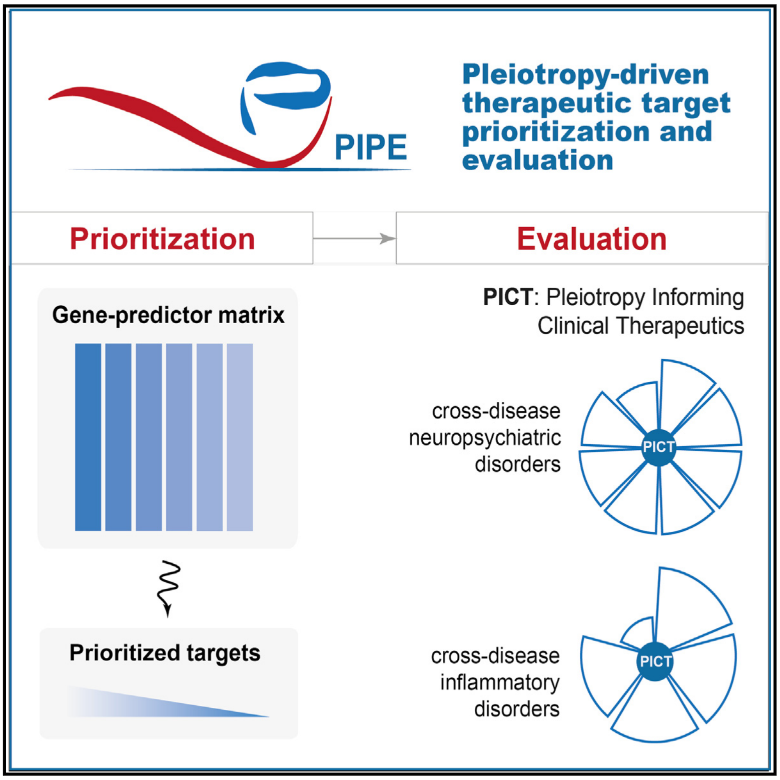

Authors

Chaohui Bao, Tingting Tan, Shan Wang, ..., Liye Chen, Haitao Lv, Hai Fang

Correspondence

liye.chen@ndorms.ox.ac.uk (L.C.),
haitaolyu@hkbu.edu.hk (H.L.),
fh12355@rjh.com.cn (H.F.)

In brief

Bao et al. introduce a pleiotropy-driven approach that leverages shared genetic and pathway architectures of related diseases to advance therapeutic discovery from genetic association studies. By applying it to inflammatory and neuropsychiatric diseases, they demonstrate its performance for *in silico* early-stage therapeutic target discovery.

Highlights

- An approach for pleiotropy-driven target prioritization and evaluation from GWASs
- The approach prioritizes therapeutic gene targets based on cross-disease genetic associations
- The approach also enables insights into shared, pathway-based mechanisms
- Performance over state-of-the-art approaches highlights the value of leveraging pleiotropy

Bao et al., 2024, Cell Reports Methods *4*, 100757
April 22, 2024 © 2024 The Author(s). Published by Elsevier Inc.
https://doi.org/10.1016/j.crmeth.2024.100757

Cell Reports Methods

CellPress
OPEN ACCESS

Article

A cross-disease, pleiotropy-driven approach for therapeutic target prioritization and evaluation

Chaohui Bao,[1,8] Tingting Tan,[1,8] Shan Wang,[1,8] Chenxu Gao,[1] Chang Lu,[2] Siyue Yang,[1,3] Yizhu Diao,[4] Lulu Jiang,[5] Duohui Jing,[1] Liye Chen,[6,*] Haitao Lv,[1,7,*] and Hai Fang[1,9,*]
[1]Shanghai Institute of Hematology, State Key Laboratory of Medical Genomics, National Research Center for Translational Medicine at Shanghai, Ruijin Hospital, Shanghai Jiao Tong University School of Medicine, Shanghai 200025, China
[2]MRC London Institute of Medical Sciences, Imperial College London, W12 0HS London, UK
[3]Faculty of Medical Laboratory Science, Ruijin Hospital, Shanghai Jiao Tong University School of Medicine, Shanghai 200025, China
[4]College of Finance and Statistics, Hunan University, Changsha, Hunan 410079, China
[5]Translational Health Sciences, University of Bristol, BS1 3NY Bristol, UK
[6]Nuffield Department of Orthopaedics, Rheumatology and Musculoskeletal Sciences, University of Oxford, OX3 7LD Oxford, UK
[7]School of Chinese Medicine, State Key Laboratory of Environmental and Biological Analysis, Hong Kong Chinese Medicine Phenome Research Center, Hong Kong Baptist University, Hong Kong 999077, China
[8]These authors contributed equally
[9]Lead contact
*Correspondence: liye.chen@ndorms.ox.ac.uk (L.C.), haitaolyu@hkbu.edu.hk (H.L.), fh12355@rjh.com.cn (H.F.)
https://doi.org/10.1016/j.crmeth.2024.100757

MOTIVATION Diseases, despite distinct clinical manifestations, may share common pathogenic mechanisms, offering opportunities for shared therapeutics. Cross-disease genome-wide association studies (GWASs) help identify pleiotropic loci, often in the non-coding genome, exerting pleiotropic effects across diseases. However, linking these pleiotropic non-coding loci to target genes and pathways poses the "*W-H-W*" challenge—whether pleiotropy aids therapeutic discovery, how to evaluate it, and in which diseases it informs clinical therapeutics. Moreover, the *status quo* approaches, such as Priority index and Open Targets, face limitations in translating findings from cross-disease GWASs into drug target prediction. Therefore, we introduce a pleiotropy-driven approach (and its R package) with the aim of providing an integrated solution to bridge the gap between pleiotropy studies and therapeutics discovery.

SUMMARY

Cross-disease genome-wide association studies (GWASs) unveil pleiotropic loci, mostly situated within the non-coding genome, each of which exerts pleiotropic effects across multiple diseases. However, the challenge "*W-H-W*" (namely, whether, how, and in which specific diseases pleiotropy can inform clinical therapeutics) calls for effective and integrative approaches and tools. We here introduce a pleiotropy-driven approach specifically designed for therapeutic target prioritization and evaluation from cross-disease GWAS summary data, with its validity demonstrated through applications to two systems of disorders (neuropsychiatric and inflammatory). We illustrate its improved performance in recovering clinical proof-of-concept therapeutic targets. Importantly, it identifies specific diseases where pleiotropy informs clinical therapeutics. Furthermore, we illustrate its versatility in accomplishing advanced tasks, including pathway crosstalk identification and downstream crosstalk-based analyses. To conclude, our integrated solution helps bridge the gap between pleiotropy studies and therapeutics discovery.

INTRODUCTION

Computational medicine, though lacking a precise definition, is increasingly recognized for its rapid facilitation of bridging the gap between the discovery of cross-disease pleiotropy and the application of translational therapeutics. This trend aligns with the evolving focus of human genetics research and drug development (R&D), which is progressively shifting toward translational use (of genetic findings), integrative prioritization (together with performance evaluation), and cross-disease analyses

© 2024 The Author(s). Published by Elsevier Inc.
This is an open access article under the CC BY license (http://creativecommons.org/licenses/by/4.0/).

(based on population-scale cohorts with rich phenotypes). It has been over 15 years since the 2007 landmark genome-wide association studies (GWASs) in common chronic diseases,[1,2] and we find ourselves in an era of "trying and testing" within human genetics R&D, as highlighted in Alzheimer's disease[3] and type 2 diabetes.[4] Despite notable advancements, the drug attrition rate remains high, with estimates suggesting that more than 90% of phase 1 clinical trial drug candidates fail to progress through the drug discovery pipeline.[5] This underscores the urgent need to increase the success rate along the pipeline. Human genetics R&D, particularly in the post-GWAS translational medicine era, is poised to play a pivotal role in addressing this challenge, marked by the three aforementioned shifts, which will be explained in greater detail below.

The first and foremost shift involves moving the emphasis from merely understanding the genetics of disease to the translational use of genetic findings.[6–8] This shift is motivated by retrospective analyses revealing that human genetic evidence supporting drug-target pairs significantly enhances the success rate of drug development,[9–11] especially when drugs are supported by targets with a causal relation to the disease.[12]

The second shift involves the development of drug-target integrative prioritization approaches, encompassing two key recipes: (1) the utilization of multimodal genomic datasets on gene regulation and (2) the incorporation of network knowledge on protein interactions. In Recipe 1, the use of regulatory genomic datasets addresses the intrinsic difficulty in linking genetic loci, mostly non-coding in common diseases, to effector genes and pathways. The regulatory effects of non-coding loci on distant genes are often exerted over considerable distances and in a cell-type-specific manner. These effects can be measured quantitively. For example, promoter-capture Hi-C studies map long-range physical interactions with gene promoters, while expression quantitative trait locus (eQTL) studies map genetic associations with gene expression. Recipe 2 emphasizes the integration of knowledge about protein interactions. This integration is motivated by the recognition that combining human genetic findings with protein interaction data significantly enhances the recovery of known therapeutic drug targets.[13,14]

The most fundamental is the shift from single-disease associations to cross-disease analyses, which was made possible by population-scale genomic and phenomic data,[15] particularly the introduction of phenomics[16] and translational phenomics.[17] Appreciation is growing for the simultaneous analysis of a broad spectrum of diseases, recognizing its potential to boost the discovery in genetics. Cross-disease analyses have proven instrumental in identifying shared genetic associations, each exhibiting pleiotropic effects across multiple diseases. This cross-disease analysis has found success in the investigation of neuropsychiatric disorders[18] and inflammatory disorders.[19] The term pleiotropic originates from the Greek words "pleio" (many) and "tropic" (ways). In the context of disease genetics, pleiotropy refers to a phenomenon where a genetic locus or single gene influences multiple traits.[20] Specifically, the term "pleiotropic loci" denotes shared genetic loci each influencing two or more diseases. This concept differs from "polygenic loci," which describes multiple genetic loci converging to impact a single disease. Remarkably, pleiotropic effects manifest in diseases with often divergent phenotypes or distinct clinical manifestations. This aligns with Mendel's study of inheritance in pea plants a century ago observing that the "seed coat color" gene affected apparently unrelated traits, such as flower and axil pigmentation.

Diseases with diverse phenotypes, such as neuropsychiatric disorders, may share underlying pathogenic mechanisms, presenting significant opportunities for therapeutic sharing. Identifying shared pleiotropic loci and elucidating their effector genes and pathways hold profound implications for therapeutic target discovery. The central question arising is how to effectively identify therapeutic targets from cross-disease pleiotropic associations. We can learn from what is already known, particularly insights from the two aforementioned recipes when developing integrative approaches primarily for individual diseases. Alongside contributions from others,[21] our work positions us at the forefront of developing integrative prioritization approaches and tools tailored for individual diseases, such as Alzheimer's disease,[22] ankylosing spondylitis,[23] asthma,[24] critical COVID-19,[25] endometriosis,[26] immunoglobulin A nephropathy,[27] kidney stone disease,[28] myasthenia gravis,[29,30] and type 1 diabetes.[31] These tools harness human disease genetics in conjunction with a wealth of functional genomic datasets aimed at facilitating early-stage therapeutic target discovery. The principle of genetics-led target discovery is gaining increasing recognition and is being employed to enhance late-stage drug approval processes.

In this study, we establish that this principle is also applicable to cross-disease pleiotropic associations. This is demonstrated by the development of a pleiotropy-driven approach, pleiotropy informing prioritization and evaluation (or PIPE), designed to enable pleiotropy-driven therapeutic target prioritization and evaluation (Figure 1). We substantiate the validity of PIPE by showcasing its improved performance in generating therapeutic target resources for neuropsychiatric and inflammatory disorders. We also show the potential of PIPE in: (1) identifying target genes involved in pathway crosstalk; (2) revealing functional modules with therapeutic potential and evolutionary origin, particularly co-arising of inflammatory disorder-specific modules with the speciation of bony fish; and (3) constructing a prioritization map in which structurally targetable calcium signaling genes are specific to neuropsychiatric disorders. Alongside freely accessible resources and step-by-step reproducible showcases, PIPE represents a valuable tool for *in silico* early-stage therapeutic target discovery, with a specific focus on pleiotropic evidence.

RESULTS

In this study, we aimed to determine whether the principle and two recipes outlined earlier are also applicable to cross-disease pleiotropic association summary data. Specifically, we sought to address the "*W-H-W*" challenge: (1) whether pleiotropic associations are useful for therapeutic target discovery, (2) how to implement and evaluate target identification based on pleiotropic evidence, and (3) in which specific diseases pleiotropy can inform clinical therapeutics. To tackle this challenge, we developed a pleiotropy-driven approach, "PIPE," with further details provided in the subsequent subsection.

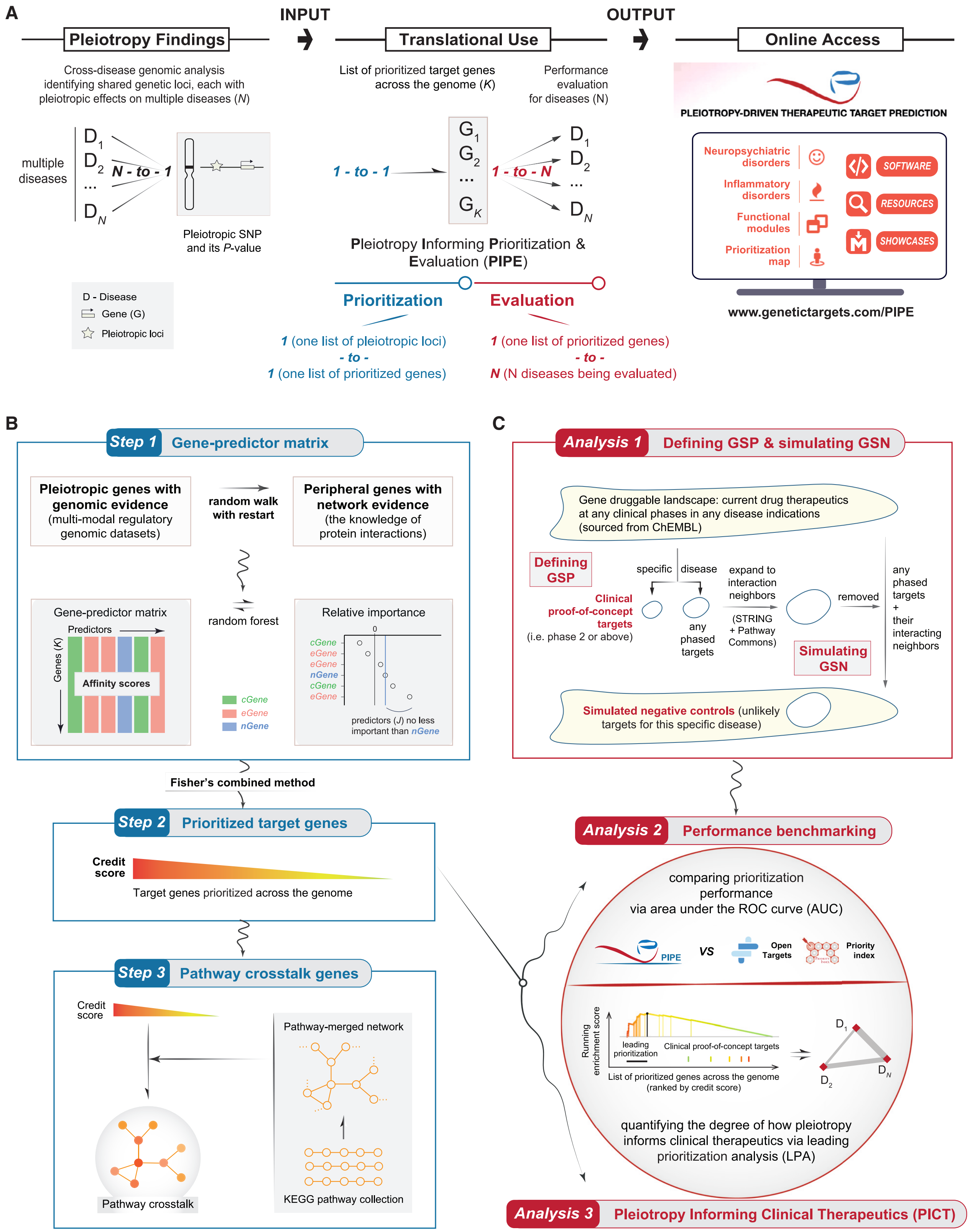

(legend on next page)

Establishment of PIPE for therapeutic target prioritization and evaluation

As indicated by its name, PIPE features two components — prioritization and evaluation. It excels in *ab initio* resolving pleiotropic associations derived from cross-disease genetic findings, facilitating therapeutic target prioritization and evaluation, and generating accessible online resources (Figure 1A). To achieve this, we devised three prioritization steps (Figure 1B) and three evaluation analyses (Figure 1C).

Using pleiotropic associations as inputs, PIPE systematically runs through three sequential steps to output therapeutic targets at both the gene and pathway crosstalk levels (Figure 1B; STAR Methods). A distinctive feature of PIPE is its capacity to establish the link from pleiotropic loci, including those located within the non-coding genome, to candidate target genes. This is made possible by harnessing the value of multimodal regulatory genomic datasets (genomic evidence) and leveraging knowledge of protein interactions (network evidence).

Accordingly, at Step 1, right from pleiotropic associations, we identified pleiotropic genes supported by genomic evidence, termed "pleiotropic (genomic) genes." These encompassed three types (or predictors): nearby genes (*nGenes*) with evidence of genomic proximity, conformation genes (*cGenes*) identified using promoter-capture Hi-C datasets,[32,33] and expression-associated genes (*eGenes*) identified using eQTL datasets.[34] For each type (or per dataset) of pleiotropic genes, we further identified peripheral genes supported by network evidence, termed "peripheral (networked) genes," identified so through utilizing high-quality protein interaction data.[35] It is noteworthy that only one *nGene* predictor was prepared, while the number of *cGene* predictors (or *eGene* predictors) equaled the number of datasets used (i.e., one predictor per dataset). We then assessed the relative importance of predictors, retaining subsets of *cGene* and *eGene* predictors that were at least as important as the *nGene* predictor (conventionally used as the baseline predictor). This process enabled the extraction of informative predictors, subsequently forming a gene-predictor matrix. Similar to Fisher's meta-analysis combining predictors in the gene-predictor matrix, at Step 2, we generated a comprehensive list of prioritized target genes, each assigned a credit score on a 10 point scale. Moving to Step 3, from this compilation of all prioritized target genes, we identified a concise and manageable list of genes likely mediating the crosstalk between molecular pathways, representing targets at the pathway crosstalk level.

To evaluate the performance of PIPE in target gene prioritization, we designed three instrumental analyses (Figure 1C; STAR Methods). Firstly, we outlined the supportive procedures for defining clinical proof-of-concept targets (phase 2 or higher) for a specific disease and simulating negative controls that are unlikely targets for this disease. Secondly, we benchmarked PIPE against the state-of-the-art prioritization approach, namely Open Targets[36,37]—the most relevant approach that also utilizes human genetics and genomics for target identification and validation. This benchmarking was measured by the area under the ROC curve (AUC), assessing the ability to distinguish clinical proof-of-concept targets from simulated negative targets. Thirdly, we introduced a new metric, called "pleiotropy informing clinical therapeutics (PICT)," to quantify the tendency of prioritized genes to be clinical proof-of-concept targets via leading prioritization analysis (LPA).

Validating the utility of PIPE in addressing the "*W-H-W*" challenge for two systems of disorders

First, we evaluated the effectiveness of PIPE in predicting therapeutic targets from cross-disease pleiotropic associations, focusing on neuropsychiatric disorders that encompassed eight specific diseases.[18] As illustrated in Figures 2A and S1A, our benchmarking strongly supported the validity of PIPE in predicting therapeutic targets, especially when compared to Open Targets,[37] particularly its Genetics Portal.[36] Regarding Open Targets, prioritizations based on individual evidence were used for evaluation, namely, genetic association and text mining, both obtained from the Open Targets Platform.[37] Despite being purely pleiotropy-driven, PIPE outperformed Open Targets (genetic association) in all diseases analyzed and demonstrated competitiveness with Open Targets (text mining) for most diseases. Subsequently, we performed LPA to quantify the PICT potential for each specific disease (Figure 2B; Table S1). LPA assesses the extent to which clinical proof-of-concept targets are enriched at the "leading prioritization" of the entire prioritized gene list and reports three pieces of information on the enrichment results: normalized enrichment score, enrichment significance (false discovery rate), and the fraction of clinical proof-of-concept targets recovered at the leading prioritization (coverage). All three results were factored into the calculation of PICT. This enabled the identification of specific diseases in which pleiotropy can inform clinical therapeutics. The highest PICT was observed for attention-deficit hyperactivity disorder, with its leading prioritization plot presented in Figure 2B (top right), where clinical proof-of-concept targets are explicitly labeled.

Next, we demonstrated the utility of PIPE in elucidating inter-disease relationships within neuropsychiatric disorders (Figures 2C and 2D) based on the shared prioritization of therapeutic targets. For each disease, we defined its member genes as clinical proof-of-concept targets at the leading prioritization. The relationship between any two diseases was inferred by the extent of sharing their member genes (Figure 2C). As shown in

Figure 1. Overview of pleiotropy informing prioritization and evaluation (PIPE)
(A) Conceptual illustration. The artwork PIPE is designed to resemble a computational framework for pleiotropy-driven therapeutic target prioritization and evaluation.
(B) Three steps designed for therapeutic target prioritization at both the gene and pathway crosstalk levels. Notably, *nGene* (in blue) refers to nearby genes with evidence of genomic proximity, *cGene* (in green) refers to conformation genes with evidence of gene promoters physically interacting with SNP-harboring genomic regions, and *eGene* (in pink) refers to expression-associated genes with evidence of genetic regulation of gene expression.
(C) Three instrumental analyses enabling performance evaluation. They include the supportive procedure of how to define clinical proof-of-concept targets and how to simulate negative controls, performance benchmarking, and pleiotropy informing clinical therapeutics (PICT) via leading prioritization analysis (LPA).

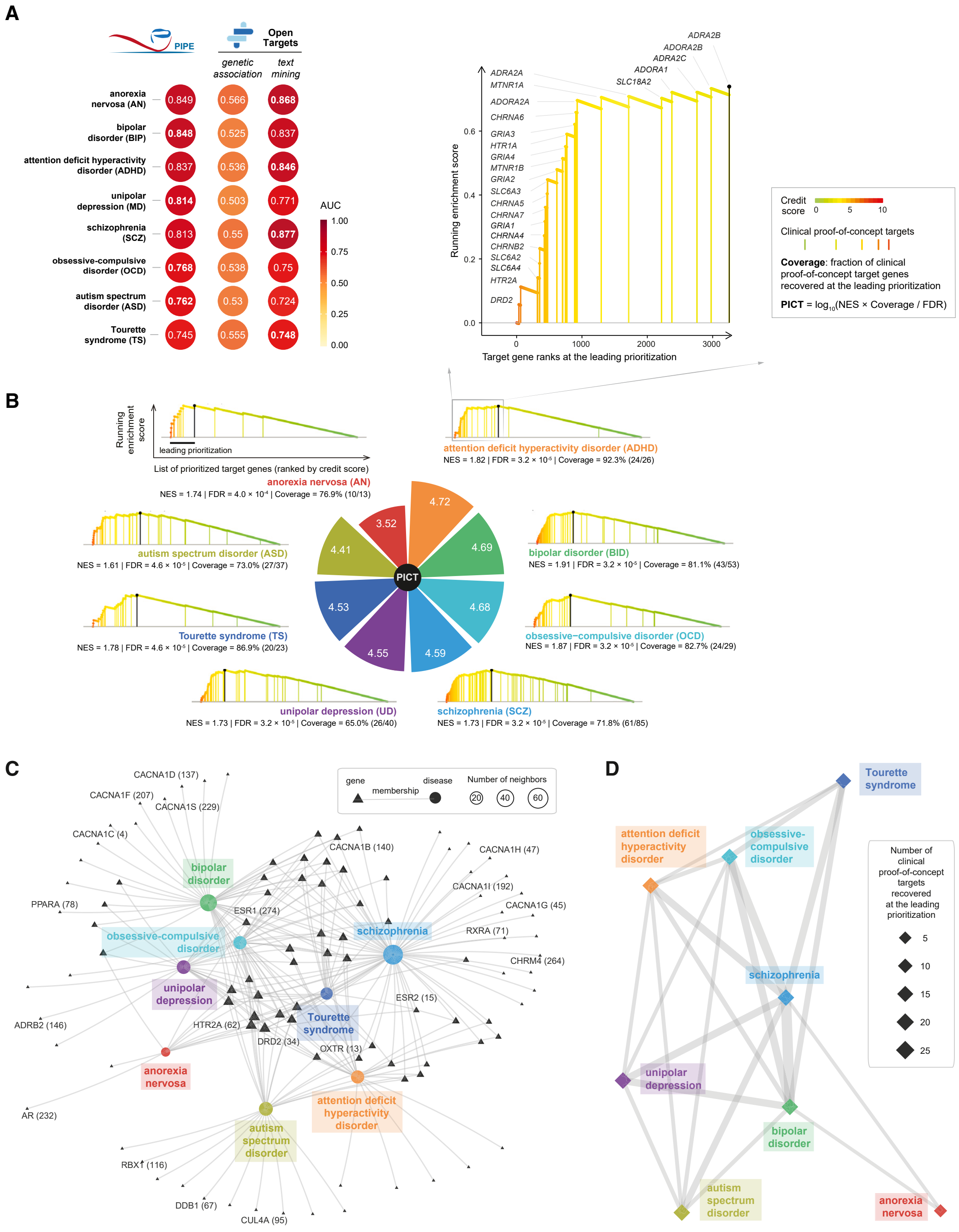

Figure 2. Performance evaluation of PIPE in recovering therapeutic targets for neuropsychiatric disorders and its utility in determining target-level disease relationships

(A) Heatmap-like performance comparisons between PIPE and Open Targets. The performance is measured by the area under the ROC curve (AUC), which separates clinical proof-of-concept targets from simulated negative targets.

(B) LPA for eight neuropsychiatric disorders. Individual diseases are arranged in clockwise order using the pie chart sized by PICT. Each leading prioritization plot presents the normalized enrichment score (NES), enrichment significance (false discovery rate [FDR]), and the fraction of clinical proof-of-concept targets found at the leading prioritization (coverage). All three variables contribute to the calculation of disease-specific PICT. The leading prioritization is defined as the leftmost

(legend continued on next page)

Figure 2D, based on the degree of shared prioritized target genes, the strongest relationships were observed among three specific neuropsychiatric diseases that impact the mind—bipolar disorder, schizophrenia, and unipolar depression. To visually represent the inferred relationships, we provided a side-by-side summary of how PIPE recovered clinical proof-of-concept targets for each disease within the neuropsychiatric disorder system (Figure 3; Table S2). Notably, approved drug targets are highlighted in bold in Figures 3B and 3C, suggesting potential opportunities for drug repurposing within neuropsychiatric disorders.

Moreover, we substantiated the validity and utility of PIPE by analyzing cross-disease pleiotropic associations for inflammatory disorders encompassing five specific diseases. Our results demonstrated the superior performance of PIPE across all specific diseases analyzed (Figures 4A and S1B). Notably, the highest PICT was seen for psoriasis and inflammatory bowel disease (Figure 4B; Table S3). Inflammatory bowel disease can be subcategorized into Crohn's disease (CRO) and ulcerative colitis (UC; Figure 4C). Consistent with clinical distinctions, we observed a close relationship between CRO and UC, which exhibited the strongest inferred connection based on the number of shared member genes (Figures 4D, 4E, and S2). Comparing PIPE to our previous Pi approach,[38,39] which concentrated on single-disease GWASs, highlighted the superior performance of PIPE (Figure 4F). This underscores the added value of pleiotropy studies, particularly employing pleiotropy approaches like PIPE, in enhancing target prioritization. To elucidate the impact of different types of PIPE predictors on prioritization, we also assessed their relevant importance, with *cGene* emerging as the most important predictor, followed by *eGene* and *nGene* (Figure 4G). This finding aligns with our previous findings from the Pi approach.[38,39]

Taken together, our results strongly validate the utility of PIPE in addressing the "*W-H-W*" challenge, providing a robust framework for therapeutic target prioritization and evaluation for both neuropsychiatric and inflammatory disorders. The validity of PIPE also motivates further exploration into its advanced utilities in therapeutic discovery.

Advanced utilities of PIPE in achieving complicated tasks on therapeutics discovery

We used Manhattan plots to illustrate a comprehensive list of target genes prioritized across the genome (Figure S3A). Additionally, we identified the most significant pathways wherein highly prioritized genes act coordinately. They included circadian entrainment and GABAergic/serotonergic synapses for neuropsychiatric disorders[40,41] and tumor necrosis factor (TNF), NOD-like receptor, and nuclear factor κB (NF-κB) signaling for inflammatory disorders[42,43] (Figure S3B). These significant pathways were consistently identified using molecular hallmarks as well (Figure S3C). Beyond individual pathways, PIPE is also unique in identifying a network of highly prioritized genes that mediate crosstalk between molecular pathways, generating a concise and manageable list of target genes at the pathway crosstalk level. To achieve this, we integrated the prioritization information with gene interactions (merged from KEGG pathways; illustrated in step 3 of Figure 1B). As shown in Figure 5 (also see Table S4), we identified a 57-gene pathway crosstalk in neuropsychiatric disorders ($p = 1.77 \times 10^{-60}$ on the permutation test) and a 59-gene pathway crosstalk in inflammatory disorders ($p = 1.39 \times 10^{-181}$ on the permutation test).

Next, we delved into unraveling the modular structure of the network by merging the identified instances of two pathway crosstalks. This exploration revealed five distinct modules (M1–M5) inherent in the network, identified so without presumptive prior knowledge. These modules were intricately associated with discernible molecular pathways (Figures 6A and S4A; Table S5), signifying a functional modular design. Among them, two modules (M3 and M5) exhibited links with inflammatory pathways, including JAK-STAT signaling with M3, TNF, and NF-κB signaling with M5. Additionally, three modules were characterized by pathways relevant to neuropathology, including cAMP and cGMP-PKG signaling (M1), cAMP and calcium signaling (M2), and apelin and PI3K-ATK signaling (M4).

When modules were color-coded by credit scores (Figure 6B), an intriguing pattern emerged. Genes in M3 and M5 generally exhibited higher credit scores in inflammatory disorders, indicating inflammatory-disorder-specific modules. In contrast, genes in M1, M2, and M4 showcased higher credit scores for neuropsychiatric disorders, signifying neuropsychiatric-disorder-specific modules (Figure 6B). Moreover, our analysis revealed a high degree of support from both preclinical evidence (non-approved phased drugs) and clinical evidence (approved drugs) for inflammatory-disorder-specific modules (Figure 6C). However, support from clinical evidence, albeit not from preclinical evidence, was notable for neuropsychiatric-disorder-specific modules (Figure 6C). Collectively, our investigation of pathways and modules for targets prioritized from pleiotropy GWASs unveiled a notable separation between neuroscience and immunological diseases. While acknowledging that GWAS loci tend to concentrate in specific genomic locations irrespective of the trait, we underscore that such concentration does not necessarily imply shared mechanisms. Our findings underscore the significance of further exploring the divergence or convergence of pathways/modules across different traits, adding a layer of complexity and relevance to the understanding of the molecular basis of complex diseases.

We hypothesized that disorder-specific functional modules might bear an evolutionary origin. To test this hypothesis, we traced the evolutionary history of gene creation, denoted as

region ahead of the peak (the dark bar). Inserted at the top right is the leading prioritization plot for attention-deficit hyperactivity disorder (ADHD), where recovered clinical proof-of-concept targets are indicated by vertical lines, labeled by gene symbols, and color coded by the credit score.

(C) Network-like representation of diseases and member genes (i.e., clinical proof-of-concept targets recovered at the leading prioritization). Member genes shared between any two diseases are also labeled, including target ranks (in the parentheses). The network layout was rendered using the stress majorization algorithm.

(D) Inter-disease relationships inferred by the sharing of member genes. Nodes are sized by the number of member genes, and edges represent relationships (with the thickness proportional to the number of genes shared between two endpoint diseases).

See also Figure S1 and Table S1.

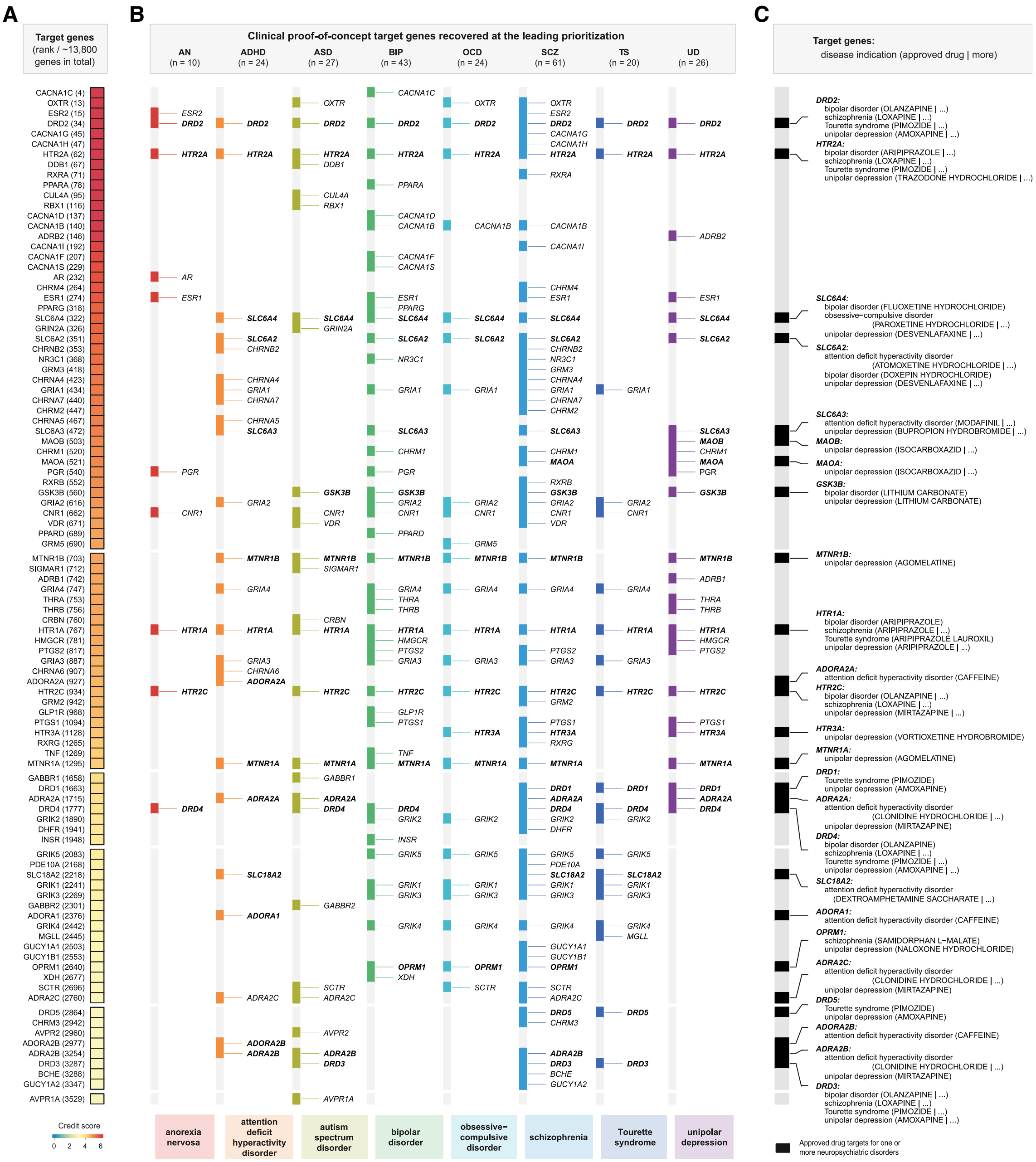

Figure 3. Illustration of clinical proof-of-concept therapeutic targets recovered at the leading prioritization for each individual disease within neuropsychiatric disorders

(A) Heatmap for clinical proof-of-concept therapeutic targets. The credit score is color coded, with the number in the parentheses indicating the target rank.
(B) Annotations to clinical proof-of-concept targets per disease.
(C) Annotations for approved drug targets, disease indications, and approved drugs.
See also Figure S1 and Table S2.

"gene evolutionary age." For a human gene, its evolutionary age is defined as the phylostratum in which it first emerged, and gene creation events were dated through genomic phylostratigraphy.[44] Our investigation revealed that genes within inflammatory disorder-specific modules tended to originate in *Bilateria*, especially in its descendant *Euteleostomi* (or "bony fish"), coinciding with the emergence of genes involved in NF-κB and JAK-STAT signalings (Figures 6D and S4B). This co-emergence aligns with the findings from evolutionary analysis for immune system genes in teleost/bony fishes.[45] In contrast, we did not find evidence supporting a preferential gene creation pattern for neuropsychiatric disorder-specific modules.

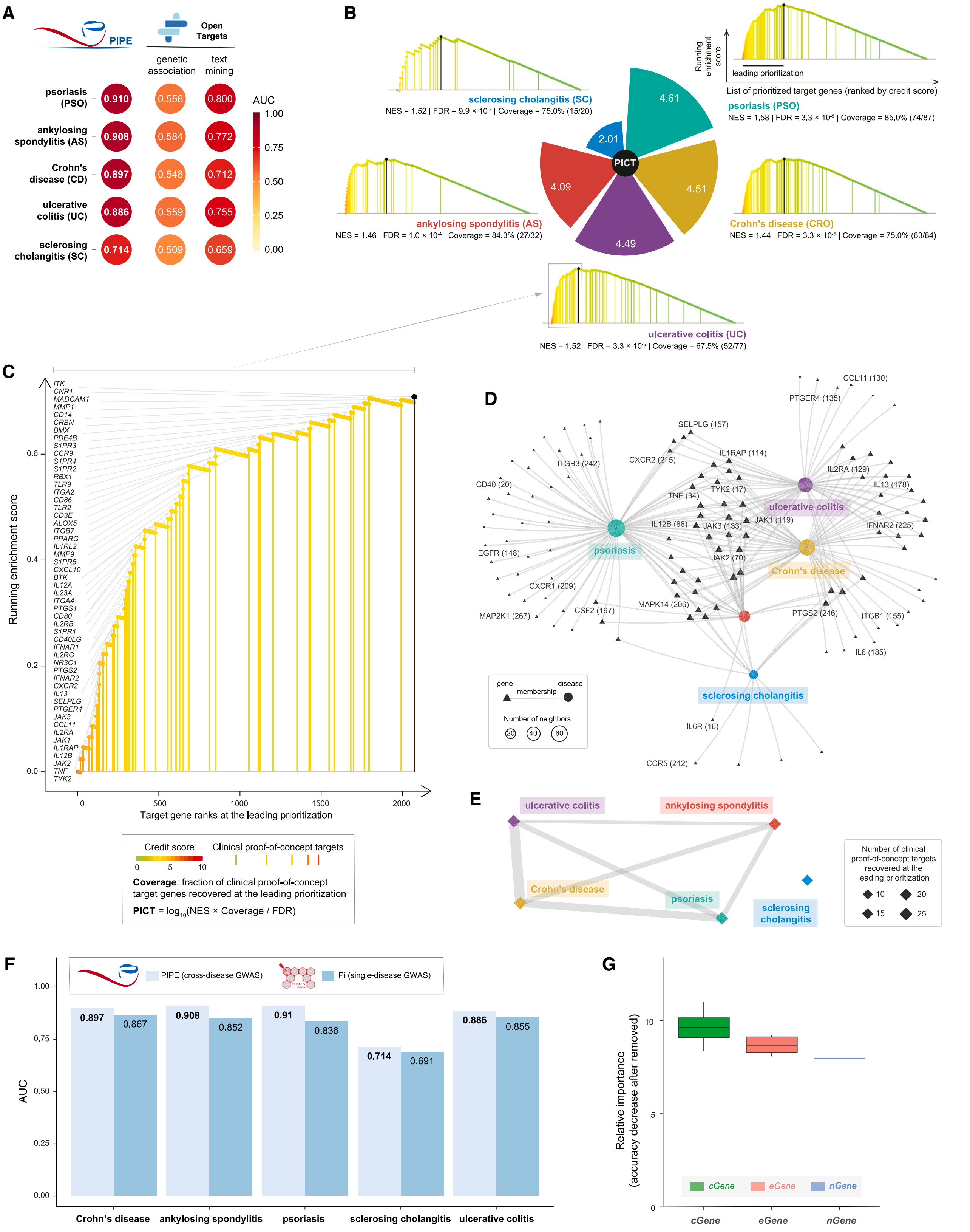

(legend on next page)

Finally, focusing on the genes implicated in two instances of pathway crosstalks, we employed a supra-hexagonal map[46–50] to construct a prioritization map (Figures 7A and S5A). The trained map was color-coded separately for neuropsychiatric and inflammatory disorders (Figures 7B and S5B). We grouped adjacent hexagons in a topology-preserving manner, leading to the identification of three target clusters (C1–C3) that exhibited both shared and distinct characteristics between the two disorder systems (Figure 7C; Table S6). Genes in cluster C1 demonstrated significantly higher credit scores in inflammatory disorders. Although genes in cluster C2 were slightly shared, they leaned toward stronger associations with inflammatory disorders. In contrast, genes in cluster C3 displayed markedly higher credit scores in neuropsychiatric disorders. Relative to the clusters specific to inflammatory disorders, the neuropsychiatric disorder-specific cluster C3 contained a notably lower proportion of structurally targetable genes (Figure 7C), indicating that these genes are under-studied. A gene was classified as targetable if its known protein structures[51–54] were predicted to contain druggable pockets. Despite genes in C3 being under-studied (i.e., lacking genes already targeted by non-approved or approved drugs), a closer inspection of the structural evidence enabled the identification of the targetable genes involved in calcium signaling (*CAMK2A*, *CAMK2D*, and *CALML3*), which were also predicted to contain druggable pockets (Figures 7D and 7E). Considering the significance of calcium/calmodulin-dependent protein kinases in synaptic plasticity and neuropsychiatric disease,[55,56] we propose that these structurally targetable calcium signaling genes hold particular interest in informing disease-specific targeting potential in neuropsychiatric disorders.

DISCUSSION

This study convincingly tackles the "*W-H-W*" challenge through the introduction of a pleiotropy-driven approach designed to resolve pleiotropic association data for translational use in therapeutic target identification and validation. By improving therapeutic target discovery, PIPE represents a substantial leap toward narrowing the gap between cross-disease pleiotropy discovery and translational therapeutic applications. Our work adds a new translational strategy for early-stage therapeutics development with a specific focus on pleiotropic evidence. This approach proves particularly valuable for diseases that, despite manifesting distinct phenotypes, share therapeutic relevance. Consequently, our efforts deepen the ongoing quest for accurate therapeutic target prediction and validation. In a broader context, PIPE advances computational medicine that leverages human disease genetics and genomics for therapeutic target discovery.

We have demonstrated the efficacy of PIPE across two paradigmatic systems—neuropsychiatric and inflammatory disorders. These systems, often employed as models for studying cross-disease genetic pleiotropy due to their high genetic heritability and therapeutic overlap, serve as robust testing grounds for the capabilities of PIPE. It is to identify target genes that mediate crosstalk between molecular pathways. Within the crosstalk network, functional modules are enriched for approved drug targets and thus offer insights into therapeutic potential. Additionally, the crosstalk-based prioritization map can reveal structurally targetable calcium signaling genes specific to neuropsychiatric disorders. The scientific community is encouraged to leverage PIPE for their own datasets, extrapolating our insights to diverse disorder systems.

Both PIPE and Open Targets rely on similar features—distance, promoter-capture Hi-C, and eQTLs—in their target prioritization processes. Consequently, a target gene supported by multiple lines of evidence is assigned with a higher priority in both approaches. Nevertheless, PIPE distinguishes itself from Open Targets in two crucial aspects. Firstly, Open Targets employs a weighted harmonic sum strategy to aggregate scores specific to individual data sources, including the locus-to-gene score from the Open Targets Genetics Portal. In contrast, PIPE calculates the credit score using Fisher's combined method applied to the gene-predictor matrix that contains affinity scores. Secondly, PIPE incorporates the ability to identify functionally linked targets with no direct genetic evidence (i.e., peripheral genes) within the context of gene interaction networks — the capability absent in Open Targets (and its Genetics Portal). These distinctions, particularly the additional use of gene interaction networks (potentially eQTL networks[57]), likely contribute to the observed improved performance over genetic association scores from Open Targets.

Limitations of the study

It should be noted, however, that PIPE is limited by the availability of regulatory genomic datasets. The use of such datasets is

Figure 4. Performance evaluation, PICT, and inter-disease relationships for inflammatory disorders

(A) Performance comparison between PIPE and Open Targets. AUC plots compare the performances that separate clinical proof-of-concept targets from simulated negative controls.

(B) Illustration of PICT for each individual disease within inflammatory disorders arranged in a clockwise order using the pie chart. Leading prioritization plot for each disease presents the NES, enrichment significance (FDR), and the fraction of clinical proof-of-concept targets found at the leading prioritization (coverage).

(C) Illustration of the leading prioritization for ulcerative colitis (UC). Clinical proof-of-concept targets are indicated by vertical lines, labeled with gene symbols, and color coded by credit scores.

(D) Network-like representation of diseases and member genes. Member genes shared between any two diseases are labeled, including target ranks (in the parentheses). The network layout was rendered using the stress majorization algorithm.

(E) Inferred inter-disease relationships based on shared member genes. Nodes are sized by the number of member genes, and the edge thickness represents the number of genes shared between two endpoint diseases. The layout is rendered using the Fruchterman-Reingold algorithm.

(F) Bar plot comparing the performance of PIPE with a single-disease prioritization approach (our previous Pi approach).

(G) Boxplot illustrating the relevant importance of predictors. Predictor importance is quantified by the decrease in accuracy when disabling/removing a predictor. Notably, removing an informative/important predictor would result in a significant decrease in accuracy. Different types of predictors are color coded.

See also Figure S2 and Table S3.

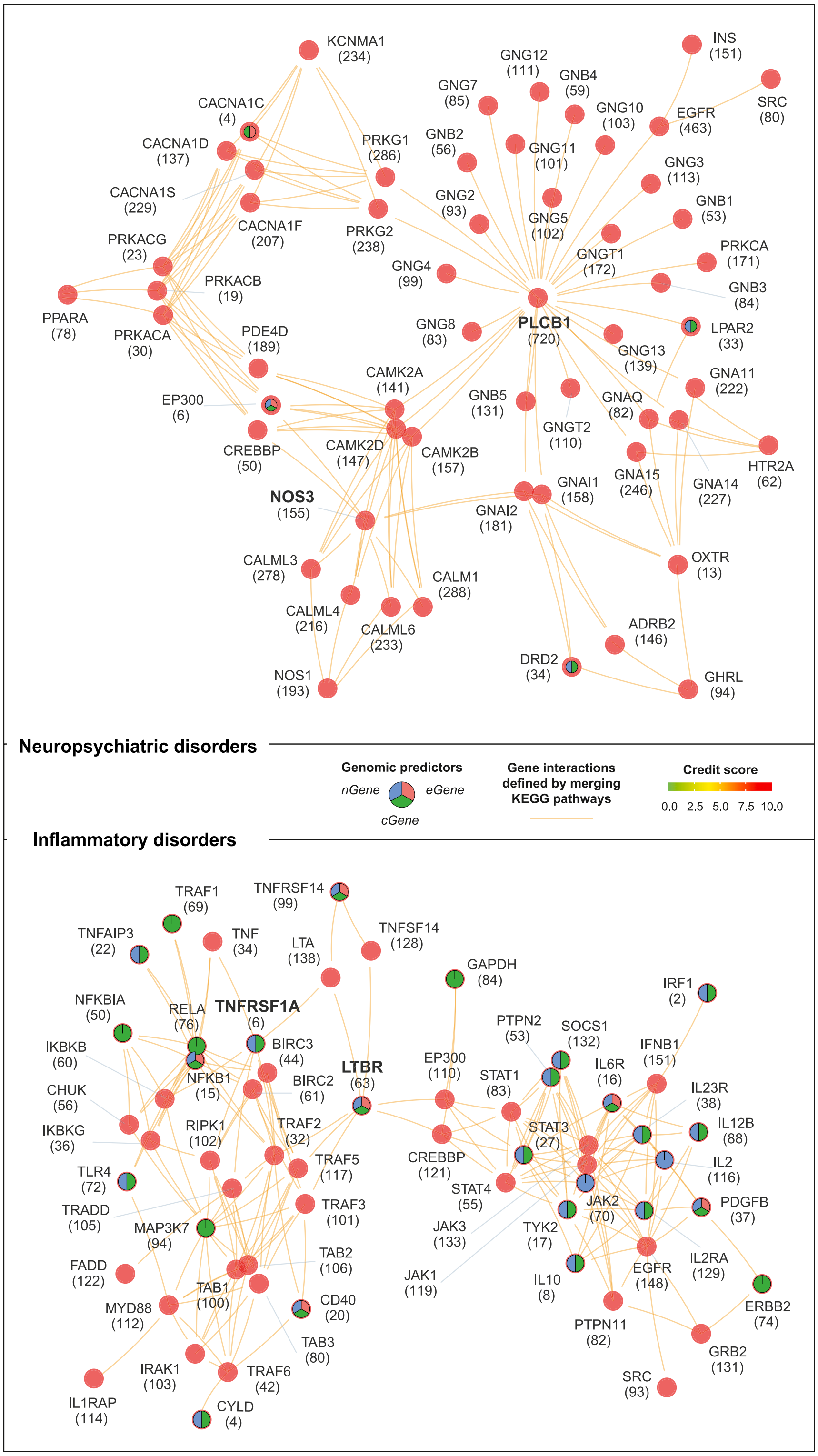

Figure 5. Identification of targets at the pathway crosstalk level for neuropsychiatric and inflammatory disorders

Visualizations of pathway crosstalks are illustrated separately for neuropsychiatric disorders (top) and inflammatory disorders (bottom). The crosstalk was identified by integrating target credit score information with pathway-derived gene interactions (in orange; merged from KEGG pathways). Nodes are labeled by gene symbols (ranks), and if identified as genomic/pleiotropic genes, they are highlighted as pie segments (with genomic predictors inside).
See also Figure S3 and Table S4.

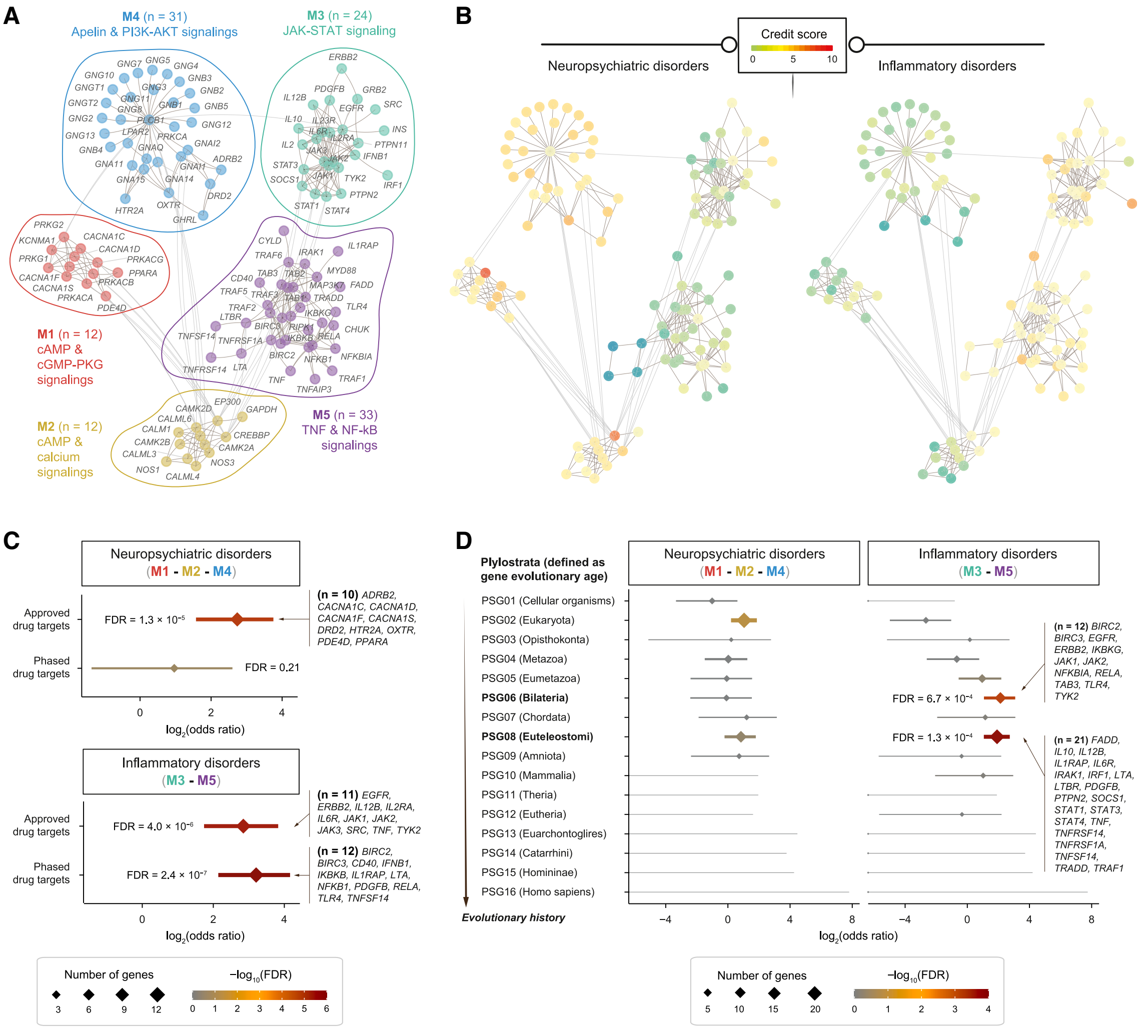

Figure 6. Modular analysis of pathway crosstalks in neuropsychiatric and inflammatory disorders

This analysis involved examining a network of gene interactions constructed by merging two instances of pathway crosstalks (as presented in Figure 5). Five modules (M1–M5) were identified through network modular analysis. Enrichment analysis was performed using a one-sided Fisher's exact test to calculate the significance level (FDR), odds ratio, and 95% confidence interval.

(A) Modular visualization of the gene network. Modules and member genes are color coded as indicated.

(B) Disorder-specific illustration of the gene network. The same network layout as in (A) is presented, with nodes color coded according to credit scores in neuropsychiatric disorders (left) and in inflammatory disorders (right).

(C) Forest plot of approved or non-approved phased drug target enrichments. Approved and non-approved phased drug targets were sourced from ChEMBL.

(D) Forest plot of phylostrata enrichments. A phylostratum contains a group of genes that were first created at that phylostratum, defined by gene evolutionary age as dated via genomic phylostratigraphy (PSG). Plylostrata are ordered according to evolutionary history from the earliest (PSG01) to the most recent (PSG16). Genes first created at each enriched phylostratum are also listed.

See also Figure S4 and Table S5.

pivotal for establishing links between non-coding loci and genomic/pleiotropic genes responsible for pleiotropic associations. Presently, the available datasets predominantly focus on immune- and brain-related contexts, thus requiring caution when extrapolating PIPE to other systems of disorders. With the increasing accessibility of promoter-capture Hi-C and eQTL datasets spanning a broader spectrum of cell types and tissues, we anticipate that pleiotropy-centered target identification and validation, via PIPE, will gain enhanced efficacy. Another potential avenue for improving target prioritization involves weighting the predictors according to their importance instead of the equal weighting implemented in this study. This alternative is particularly valuable for singling out targets, for example, supported by tissue- or cell-type-specific *eGenes*, thereby placing an emphasis on specific tissues or cell types for experimental validation. Moreover, although beyond the scope of this study, it would be potentially beneficial for future improvements to explore the inclusion of fine-mapping results in PIPE. This might involve borrowing from multidisease fine-mapping methods such as CAFEH,[58] flashfm,[59] msCAVIAR,[60] and PAINTOR,[61]

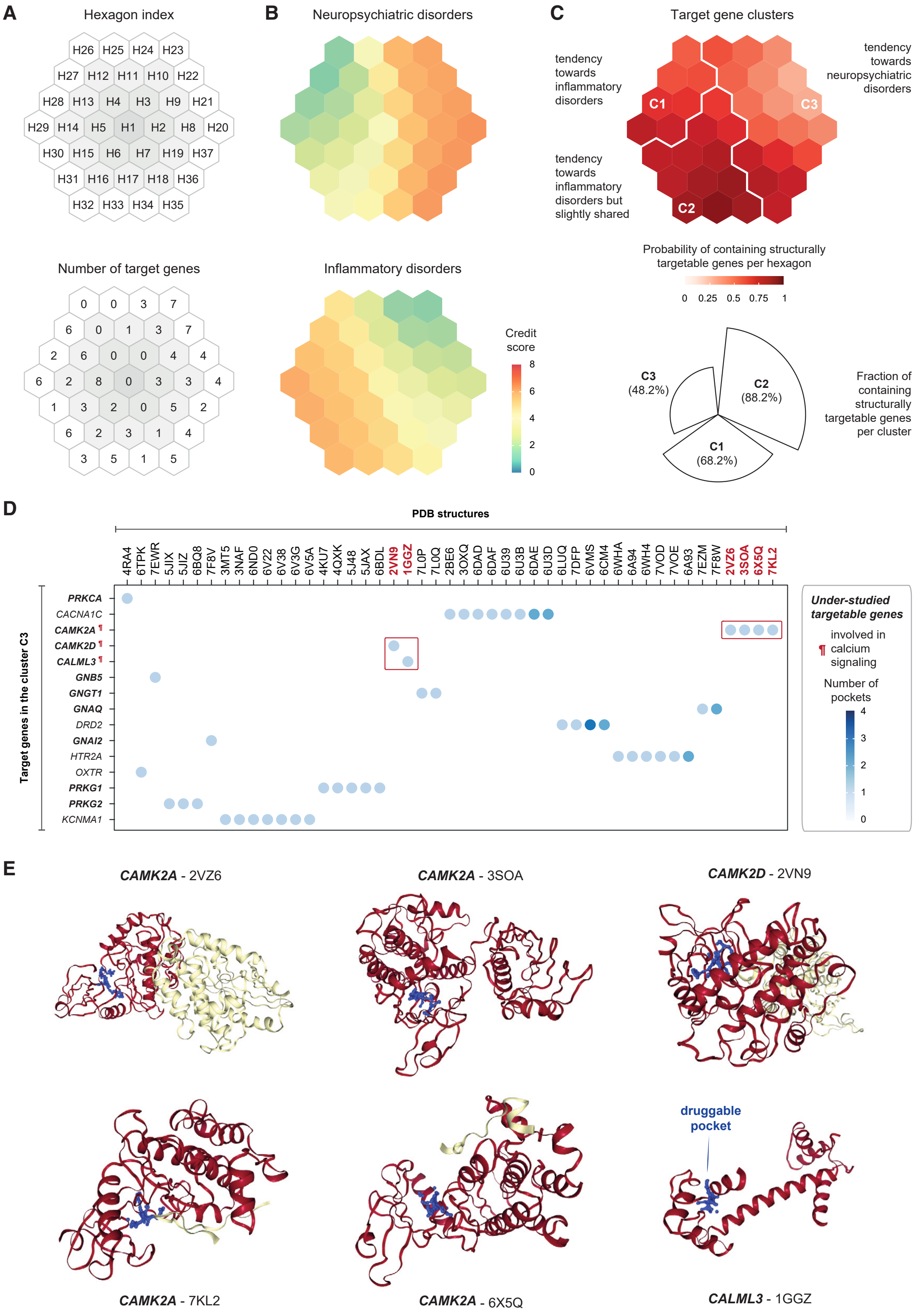

(legend on next page)

CellPress
OPEN ACCESS

which leverage summary statistics and linkage disequilibrium matrices.

STAR★METHODS

Detailed methods are provided in the online version of this paper and include the following:

- KEY RESOURCES TABLE
- RESOURCE AVAILABILITY
  - Lead contact
  - Materials availability
  - Data and code availability
- METHOD DETAILS
  - Overview of PIPE
  - The *prioritization* component of PIPE
  - The *evaluation* component of PIPE
- QUANTIFICATION AND STATISTICAL ANALYSIS
  - Crosstalk-based network modular analysis
  - Crosstalk-based prioritisation map analysis

SUPPLEMENTAL INFORMATION

Supplemental information can be found online at https://doi.org/10.1016/j.crmeth.2024.100757.

ACKNOWLEDGMENTS

We acknowledge funding from the National Natural Science Foundation of China (32170663 [H.F.] and 82300263 [C.B.]), the Shanghai Pujiang Program (21PJ1409600 [H.F.]), the Program for Professor of Special Appointment (Eastern Scholar) at Shanghai Institutions of Higher Learning (awarded to H.F.), the National Key Research and Development Program of China (2017YFC1308600 [H.L.]), the Natural Science Foundation of Shanghai (21ZR1431600 [H.L.]), the Versus Arthritis Career Development Award (22053 [L.C.]), the Academy of Medical Sciences Springboard Award (SBF0051134 [L.C.]), and the Innovative Research Team of High-Level Local Universities in Shanghai.

AUTHOR CONTRIBUTIONS

Conceptualization, H.F.; methodology, H.F., C.B., T.T., and S.W.; software, H.F.; formal analysis, H.F.; investigation, H.F., C.B., T.T., S.W., L.C., and H.L.; resources, H.F.; data curation, H.F.; writing – original draft, H.F.; writing – review & editing, C.B., T.T., S.W., C.G., C.L., S.Y., Y.D., L.J., D.J., L.C., and H.L.; visualization, H.F., C.B., and T.T.; supervision, H.F.; project administration, H.F.; funding acquisition, H.F., C.B., L.C., and H.L.

DECLARATION OF INTERESTS

The authors declare no competing interests.

Received: March 27, 2023
Revised: January 8, 2024
Accepted: March 22, 2024
Published: April 16, 2024

REFERENCES

1. Wellcome Trust Case Control Consortium (2007). Genome-wide association study of 14,000 cases of seven common diseases and 3,000 shared controls. Nature *447*, 661–678. https://doi.org/10.1038/nature05911.
2. Uffelmann, E., Huang, Q.Q., Munung, N.S., de Vries, J., Okada, Y., Martin, A.R., Martin, H.C., Lappalainen, T., and Posthuma, D. (2021). Genome-wide association studies. Nat. Rev. Methods Primers *1*, 59–21. https://doi.org/10.1038/s43586-021-00056-9.
3. Mattis, K.K., and Gloyn, A.L. (2020). From Genetic Association to Molecular Mechanisms for Islet-cell Dysfunction in Type 2 Diabetes. J. Mol. Biol. *432*, 1551–1578. https://doi.org/10.1016/j.jmb.2019.12.045.
4. Quan, M., Cao, S., Wang, Q., Wang, S., and Jia, J. (2023). Genetic Phenotypes of Alzheimer's Disease: Mechanisms and Potential Therapy. Phenomics *3*, 333–349. https://doi.org/10.1007/s43657-023-00098-x.
5. Hay, M., Thomas, D.W., Craighead, J.L., Economides, C., and Rosenthal, J. (2014). Clinical development success rates for investigational drugs. Nat. Biotechnol. *32*, 40–51. https://doi.org/10.1038/nbt.2786.
6. Reay, W.R., and Cairns, M.J. (2021). Advancing the use of genome-wide association studies for drug repurposing. Nat. Rev. Genet. *22*, 658–671. https://doi.org/10.1038/s41576-021-00387-z.
7. Fang, H., Chen, L., and Knight, J.C. (2020). From genome-wide association studies to rational drug target prioritisation in inflammatory arthritis. Lancet. Rheumatol. *2*, e50–e62. https://doi.org/10.1016/S2665-9913(19)30134-1.
8. Wang, T., Yan, Z., Zhang, Y., Lou, Z., Zheng, X., Mai, D., Wang, Y., Shang, X., Xiao, B., Peng, J., and Chen, J. (2024). postGWAS: A web server for deciphering the causality post the genome-wide association studies. Comput. Biol. Med. *171*, 108108. https://doi.org/10.1016/j.compbiomed.2024.108108.
9. Cook, D., Brown, D., Alexander, R., March, R., Morgan, P., Satterthwaite, G., and Pangalos, M.N. (2014). Lessons learned from the fate of AstraZeneca's drug pipeline: a five-dimensional framework. Nat. Rev. Drug Discov. *13*, 419–431. https://doi.org/10.1038/nrd4309.
10. Nelson, M.R., Tipney, H., Painter, J.L., Shen, J., Nicoletti, P., Shen, Y., Floratos, A., Sham, P.C., Li, M.J., Wang, J., et al. (2015). The support of human genetic evidence for approved drug indications. Nat. Genet. *47*, 856–860. https://doi.org/10.1038/ng.3314.
11. Waring, M.J., Arrowsmith, J., Leach, A.R., Leeson, P.D., Mandrell, S., Owen, R.M., Pairaudeau, G., Pennie, W.D., Pickett, S.D., Wang, J., et al.

Figure 7. Crosstalk-based prioritization map for neuropsychiatric and inflammatory disorders
(A) Illustration of the supra-hexagonal map. The map was used to analyze a matrix (containing credit scores) of 112 pathway crosstalk target genes identified in either neuropsychiatric disorders or inflammatory disorders. Top: the 2D map architecture consists of 37 hexagons indexed circling outward (H1–H37). Bottom: the number of genes mapped onto each hexagon.
(B) Prioritization map showing credit score profiles for neuropsychiatric disorders (top) and inflammatory disorders (bottom).
(C) Target gene clusters. The prioritization map was divided into 3 clusters (C1–C3), each covering continuous hexagons separated by white borders. Each hexagon is color coded by the probability of containing structurally targetable genes. The percentage of targetable genes per cluster is also shown below (polar bar). A gene was defined as targetable if it was predicted to have drug-like binding sites (i.e., druggable pockets) based on its known protein structure(s).
(D) Druggable pockets for genes in C3 specific for neuropsychiatric disorders. The dot plot presents 15 targetable genes (y axis) and their PDB known protein structures (x axis). The number of druggable pockets predicted based on PDB structures is color coded. Highlighted in bold are structurally targetable genes, which are also marked if involved in calcium signaling.
(E) Illustration of druggable pockets viewed in 3D within PDB structures for calcium signaling genes in C3. For each structure, a druggable pocket is illustrated using 3D blue balls.
See also Figure S5 and Table S6.

(2015). An analysis of the attrition of drug candidates from four major pharmaceutical companies. Nat. Rev. Drug Discov. *14*, 475–486. https://doi.org/10.1038/nrd4609.

12. King, E.A., Davis, J.W., and Degner, J.F. (2019). Are drug targets with genetic support twice as likely to be approved? Revised estimates of the impact of genetic support for drug mechanisms on the probability of drug approval. PLoS Genet. *15*, e1008489. https://doi.org/10.1371/journal.pgen.1008489.
13. Barrio-Hernandez, I., Schwartzentruber, J., Shrivastava, A., Del-Toro, N., Gonzalez, A., Zhang, Q., Mountjoy, E., Suveges, D., Ochoa, D., Ghoussaini, M., et al. (2023). Network expansion of genetic associations defines a pleiotropy map of human cell biology. Nat. Genet. *55*, 389–398. https://doi.org/10.1038/s41588-023-01327-9.
14. Okada, Y., Wu, D., Trynka, G., Raj, T., Terao, C., Ikari, K., Kochi, Y., Ohmura, K., Suzuki, A., Yoshida, S., et al. (2014). Genetics of rheumatoid arthritis contributes to biology and drug discovery. Nature *506*, 376–381. https://doi.org/10.1038/nature12873.
15. Ying, W. (2023). Phenomic Studies on Diseases: Potential and Challenges. Phenomics *3*, 285–299. https://doi.org/10.1007/s43657-022-00089-4.
16. Jin, L. (2021). Welcome to the Phenomics. Phenomics *1*, 1–2. https://doi.org/10.1007/s43657-020-00009-4.
17. Fang, H., Chen, L., Hood, L., and Tian, Q. (2023). Editorial: Translational phenomics and its applications in immunotherapy. Front. Immunol. *14*, 1211704. https://doi.org/10.3389/fimmu.2023.1211704.
18. Lee, P.H., Anttila, V., Won, H., Feng, Y.C.A., Rosenthal, J., Zhu, Z., Tucker-Drob, E.M., Nivard, M.G., Grotzinger, A.D., Posthuma, D., et al. (2019). Genomic Relationships, Novel Loci, and Pleiotropic Mechanisms across Eight Psychiatric Disorders. Cell *179*, 1469–1482.e11. https://doi.org/10.1016/j.cell.2019.11.020.
19. Ellinghaus, D., Jostins, L., Spain, S.L., Cortes, A., Bethune, J., Han, B., Park, Y.R., Raychaudhuri, S., Pouget, J.G., Hübenthal, M., et al. (2016). Analysis of five chronic inflammatory diseases identifies 27 new associations and highlights disease-specific patterns at shared loci. Nat. Genet. *48*, 510–518. https://doi.org/10.1038/ng.3528.
20. Watanabe, K., Stringer, S., Frei, O., Umićević Mirkov, M., de Leeuw, C., Polderman, T.J.C., van der Sluis, S., Andreassen, O.A., Neale, B.M., and Posthuma, D. (2019). A global overview of pleiotropy and genetic architecture in complex traits. Nat. Genet. *51*, 1339–1348. https://doi.org/10.1038/s41588-019-0481-0.
21. Mountjoy, E., Schmidt, E.M., Carmona, M., Schwartzentruber, J., Peat, G., Miranda, A., Fumis, L., Hayhurst, J., Buniello, A., Karim, M.A., et al. (2021). An open approach to systematically prioritize causal variants and genes at all published human GWAS trait-associated loci. Nat. Genet. *53*, 1527–1533. https://doi.org/10.1038/s41588-021-00945-5.
22. Schwartzentruber, J., Cooper, S., Liu, J.Z., Barrio-Hernandez, I., Bello, E., Kumasaka, N., Young, A.M.H., Franklin, R.J.M., Johnson, T., Estrada, K., et al. (2021). Genome-wide meta-analysis, fine-mapping and integrative prioritization implicate new Alzheimer's disease risk genes. Nat. Genet. *53*, 392–402. https://doi.org/10.1038/s41588-020-00776-w.
23. Brown, A.C., Cohen, C.J., Mielczarek, O., Migliorini, G., Costantino, F., Allcock, A., Davidson, C., Elliott, K.S., Fang, H., Lledó Lara, A., et al. (2023). Comprehensive epigenomic profiling reveals the extent of disease-specific chromatin states and informs target discovery in ankylosing spondylitis. Cell Genom. *3*, 100306. https://doi.org/10.1016/j.xgen.2023.100306.
24. Bao, C., Gu, L., Wang, S., Zou, K., Zhang, Z., Jiang, L., Chen, L., and Fang, H. (2023). Priority index for asthma (PIA): In silico discovery of shared and distinct drug targets for adult- and childhood-onset disease. Comput. Biol. Med. *162*, 107095. https://doi.org/10.1016/j.compbiomed.2023.107095.
25. Zhang, Z., Wang, S., Jiang, L., Wei, J., Lu, C., Li, S., Diao, Y., Fang, Z., He, S., Tan, T., et al. (2024). Priority index for critical Covid-19 identifies clinically actionable targets and drugs. Commun. Biol. *7*, 189. https://doi.org/10.1038/s42003-024-05897-0.
26. Bao, C., Wang, H., and Fang, H. (2022). Genomic evidence supports the recognition of endometriosis as an inflammatory systemic disease and reveals disease-specific therapeutic potentials of targeting neutrophil degranulation. Front. Immunol. *13*, 758440. https://doi.org/10.3389/fimmu.2022.758440.
27. Kiryluk, K., Sanchez-Rodriguez, E., Zhou, X.J., Zanoni, F., Liu, L., Mladkova, N., Khan, A., Marasa, M., Zhang, J.Y., Balderes, O., et al. (2023). Genome-wide association analyses define pathogenic signaling pathways and prioritize drug targets for IgA nephropathy. Nat. Genet. *55*, 1091–1105. https://doi.org/10.1038/s41588-023-01422-x.
28. Fang, H., and Jiang, L. (2021). Genetic prioritization, therapeutic repositioning and cross-disease comparisons reveal inflammatory targets tractable for kidney stone disease. Front. Immunol. *12*, 687291. https://doi.org/10.3389/fimmu.2021.687291.
29. Handunnetthi, L., Knezevic, B., Kasela, S., Burnham, K.L., Milani, L., Irani, S.R., Fang, H., and Knight, J.C. (2021). Genomic insights into myasthenia gravis identify distinct immunological mechanisms in early and late onset disease. Ann. Neurol. *90*, 455–463. https://doi.org/10.1002/ana.26169.
30. Chia, R., Saez-Atienzar, S., Murphy, N., Chiò, A., Blauwendraat, C., International Myasthenia Gravis Genomics Consortium; Roda, R.H., Tienari, P.J., Kaminski, H.J., Ricciardi, R., et al. (2022). Identification of genetic risk loci and prioritization of genes and pathways for myasthenia gravis: a genome-wide association study. Proc. Natl. Acad. Sci. USA *119*, e2108672119. https://doi.org/10.1073/pnas.2108672119.
31. Robertson, C.C., Inshaw, J.R.J., Onengut-Gumuscu, S., Chen, W.-M., Santa Cruz, D.F., Yang, H., Cutler, A.J., Crouch, D.J.M., Farber, E., Bridges, S.L., et al. (2021). Fine-mapping, trans-ancestral and genomic analyses identify causal variants, cells, genes and drug targets for type 1 diabetes. Nat. Genet. *53*, 962–971. https://doi.org/10.1038/s41588-021-00880-5.
32. Jung, I., Schmitt, A., Diao, Y., Lee, A.J., Liu, T., Yang, D., Tan, C., Eom, J., Chan, M., Chee, S., et al. (2019). A compendium of promoter-centered long-range chromatin interactions in the human genome. Nat. Genet. *51*, 1442–1449. https://doi.org/10.1038/s41588-019-0494-8.
33. Song, M., Yang, X., Ren, X., Maliskova, L., Li, B., Jones, I.R., Wang, C., Jacob, F., Wu, K., Traglia, M., et al. (2019). Mapping cis-regulatory chromatin contacts in neural cells links neuropsychiatric disorder risk variants to target genes. Nat. Genet. *51*, 1252–1262. https://doi.org/10.1038/s41588-019-0472-1.
34. Kerimov, N., Hayhurst, J.D., Peikova, K., Manning, J.R., Walter, P., Kolberg, L., Samoviča, M., Sakthivel, M.P., Kuzmin, I., Trevanion, S.J., et al. (2021). A compendium of uniformly processed human gene expression and splicing quantitative trait loci. Nat. Genet. *53*, 1290–1299. https://doi.org/10.1038/s41588-021-00924-w.
35. Szklarczyk, D., Kirsch, R., Koutrouli, M., Nastou, K., Mehryary, F., Hachilif, R., Gable, A.L., Fang, T., Doncheva, N.T., Pyysalo, S., et al. (2023). The STRING database in 2023: protein-protein association networks and functional enrichment analyses for any sequenced genome of interest. Nucleic Acids Res. *51*, D638–D646. https://doi.org/10.1093/nar/gkac1000.
36. Ghoussaini, M., Mountjoy, E., Carmona, M., Peat, G., Schmidt, E.M., Hercules, A., Fumis, L., Miranda, A., Carvalho-Silva, D., Buniello, A., et al. (2021). Open Targets Genetics: Systematic identification of trait-associated genes using large-scale genetics and functional genomics. Nucleic Acids Res. *49*, D1311–D1320. https://doi.org/10.1093/nar/gkaa840.
37. Ochoa, D., Hercules, A., Carmona, M., Suveges, D., Baker, J., Malangone, C., Lopez, I., Miranda, A., Cruz-Castillo, C., Fumis, L., et al. (2023). The next-generation Open Targets Platform: reimagined, redesigned, rebuilt. Nucleic Acids Res. *51*, D1353–D1359. https://doi.org/10.1093/nar/gkac1046.
38. Fang, H., ULTRA-DD Consortium; De Wolf, H., Knezevic, B., Burnham, K.L., Osgood, J., Sanniti, A., Lledó Lara, A., Kasela, S., De Cesco, S., et al. (2019). A genetics-led approach defines the drug target landscape of 30 immune-related traits. Nat. Genet. *51*, 1082–1091. https://doi.org/10.1038/s41588-019-0456-1.

39. Fang, H., and Knight, J.C. (2022). Priority index: database of genetic targets in immune-mediated disease. Nucleic Acids Res. *50*, D1358–D1367. https://doi.org/10.1093/nar/gkab994.

40. Jagannath, A., Peirson, S.N., and Foster, R.G. (2013). Sleep and circadian rhythm disruption in neuropsychiatric illness. Curr. Opin. Neurobiol. *23*, 888–894. https://doi.org/10.1016/j.conb.2013.03.008.

41. Benes, F.M., and Berretta, S. (2001). GABAergic interneurons: Implications for understanding schizophrenia and bipolar disorder. Neuropsychopharmacology *25*, 1–27. https://doi.org/10.1016/S0893-133X(01)00225-1.

42. Philpott, D.J., Sorbara, M.T., Robertson, S.J., Croitoru, K., and Girardin, S.E. (2014). NOD proteins: Regulators of inflammation in health and disease. Nat. Rev. Immunol. *14*, 9–23. https://doi.org/10.1038/nri3565.

43. Yu, H., Lin, L., Zhang, Z., Zhang, H., and Hu, H. (2020). Targeting NF-κB pathway for the therapy of diseases: mechanism and clinical study. Signal Transduct. Targeted Ther. *5*, 209. https://doi.org/10.1038/s41392-020-00312-6.

44. Trigos, A.S., Pearson, R.B., Papenfuss, A.T., and Goode, D.L. (2017). Altered interactions between unicellular and multicellular genes drive hallmarks of transformation in a diverse range of solid tumors. Proc. Natl. Acad. Sci. USA *114*, 6406–6411. https://doi.org/10.1073/pnas.1617743114.

45. Malmstrøm, M., Matschiner, M., Tørresen, O.K., Star, B., Snipen, L.G., Hansen, T.F., Baalsrud, H.T., Nederbragt, A.J., Hanel, R., Salzburger, W., et al. (2016). Evolution of the immune system influences speciation rates in teleost fishes. Nat. Genet. *48*, 1204–1210. https://doi.org/10.1038/ng.3645.

46. Fang, H., and Gough, J. (2014). supraHex: An R/Bioconductor package for tabular omics data analysis using a supra-hexagonal map. Biochem. Biophys. Res. Commun. *443*, 285–289. https://doi.org/10.1016/j.bbrc.2013.11.103.

47. Fang, H., Du, Y., Xia, L., Li, J., Zhang, J., and Wang, K. (2011). A topology-preserving selection and clustering approach to multidimensional biological data. OMICS *15*, 483–494. https://doi.org/10.1089/omi.2010.0066.

48. Zhang, J., and Fang, H. (2012). Using self-organizing maps to visualize, filter and cluster multidimensional bio-omics data. In Developments and Applications of Self-Organizing Maps, M. Johnsson, ed. (InTech), pp. 181–204. https://doi.org/10.5772/51702.

49. Fang, H., and Wang, K. (2018). Regulatory genomic data cubism. iScience *3*, 217–225. https://doi.org/10.1016/j.isci.2018.04.017.

50. Tan, Y., Jiang, L., Wang, K., and Fang, H. (2019). I3: A Self-organising Learning Workflow for Intuitive Integrative Interpretation of Complex Genetic Data. Dev. Reprod. Biol. *17*, 503–510. https://doi.org/10.1016/j.gpb.2018.10.006.

51. Burley, S.K., Bhikadiya, C., Bi, C., Bittrich, S., Chao, H., Chen, L., Craig, P.A., Crichlow, G.V., Dalenberg, K., Duarte, J.M., et al. (2023). RCSB Protein Data Bank (RCSB.org): delivery of experimentally-determined PDB structures alongside one million computed structure models of proteins from artificial intelligence/machine learning. Nucleic Acids Res. *51*, D488–D508. https://doi.org/10.1093/nar/gkac1077.

52. Fang, H. (2014). dcGOR: an R package for analysing ontologies and protein domain annotations. PLoS Comput. Biol. *10*, e1003929. https://doi.org/10.1371/journal.pcbi.1003929.

53. Fang, H., and Gough, J. (2013). dcGO: database of domain-centric ontologies on functions, phenotypes, diseases and more. Nucleic Acids Res. *41*, D536–D544. https://doi.org/10.1093/nar/gks1080.

54. Bao, C., Lu, C., Lin, J., Gough, J., and Fang, H. (2023). The dcGO domain-centric ontology database in 2023: new website and extended annotations for protein structural domains. J. Mol. Biol. *435*, 168093. https://doi.org/10.1016/j.jmb.2023.168093.

55. Nanou, E., and Catterall, W.A. (2018). Calcium Channels, Synaptic Plasticity, and Neuropsychiatric Disease. Neuron *98*, 466–481. https://doi.org/10.1016/j.neuron.2018.03.017.

56. Robison, A.J. (2014). Emerging role of CaMKII in neuropsychiatric disease. Trends Neurosci. *37*, 653–662. https://doi.org/10.1016/j.tins.2014.07.001.

57. Gaynor, S.M., Fagny, M., Lin, X., Platig, J., and Quackenbush, J. (2022). Connectivity in eQTL networks dictates reproducibility and genomic properties. Cell Rep. Methods *2*, 100218. https://doi.org/10.1016/j.crmeth.2022.100218.

58. Arvanitis, M., Tayeb, K., Strober, B.J., and Battle, A. (2022). Redefining tissue specificity of genetic regulation of gene expression in the presence of allelic heterogeneity. Am. J. Hum. Genet. *109*, 223–239. https://doi.org/10.1016/j.ajhg.2022.01.002.

59. Hernández, N., Soenksen, J., Newcombe, P., Sandhu, M., Barroso, I., Wallace, C., and Asimit, J.L. (2021). The flashfm approach for fine-mapping multiple quantitative traits. Nat. Commun. *12*, 6147. https://doi.org/10.1038/s41467-021-26364-y.

60. LaPierre, N., Taraszka, K., Huang, H., He, R., Hormozdiari, F., and Eskin, E. (2021). Identifying causal variants by fine mapping across multiple studies. PLoS Genet. *17*, e1009733. https://doi.org/10.1371/journal.pgen.1009733.

61. Kichaev, G., and Pasaniuc, B. (2015). Leveraging Functional-Annotation Data in Trans-ethnic Fine-Mapping Studies. Am. J. Hum. Genet. *97*, 260–271. https://doi.org/10.1016/j.ajhg.2015.06.007.

62. Sollis, E., Mosaku, A., Abid, A., Buniello, A., Cerezo, M., Gil, L., Groza, T., Güneş, O., Hall, P., Hayhurst, J., et al. (2023). The NHGRI-EBI GWAS Catalog: knowledgebase and deposition resource. Nucleic Acids Res. *51*, D977–D985. https://doi.org/10.1093/nar/gkac1010.

63. Zdrazil, B., Felix, E., Hunter, F., Manners, E.J., Blackshaw, J., Corbett, S., de Veij, M., Ioannidis, H., Lopez, D.M., Mosquera, J.F., et al. (2024). The ChEMBL Database in 2023: a drug discovery platform spanning multiple bioactivity data types and time periods. Nucleic Acids Res. *52*, D1180–D1192. https://doi.org/10.1093/nar/gkad1004.

64. Schmidtke, P., and Barril, X. (2010). Understanding and predicting druggability. A high-throughput method for detection of drug binding sites. J. Med. Chem. *53*, 5858–5867. https://doi.org/10.1021/jm100574m.

65. 1000 Genomes Project Consortium; Abecasis, G.R., Auton, A., Brooks, L.D., DePristo, M.A., Durbin, R.M., Handsaker, R.E., Kang, H.M., Marth, G.T., and McVean, G.A. (2012). An integrated map of genetic variation from 1,092 human genomes. Nature *491*, 56–65. https://doi.org/10.1038/nature11632.

66. Loughin, T.M. (2004). A systematic comparison of methods for combining p-values from independent tests. Comput. Stat. Data Anal. *47*, 467–485. https://doi.org/10.1016/j.csda.2003.11.020.

67. Kanehisa, M., Furumichi, M., Sato, Y., Kawashima, M., and Ishiguro-Watanabe, M. (2023). KEGG for taxonomy-based analysis of pathways and genomes. Nucleic Acids Res. *51*, D587–D592. https://doi.org/10.1093/nar/gkac963.

68. Fang, H., Knezevic, B., Burnham, K.L., and Knight, J.C. (2016). XGR software for enhanced interpretation of genomic summary data, illustrated by application to immunological traits. Genome Med. *8*, 129. https://doi.org/10.1186/s13073-016-0384-y.

69. Bao, C., Wang, S., Jiang, L., Fang, Z., Zou, K., Lin, J., Chen, S., and Fang, H. (2023). OpenXGR: a web-server update for genomic summary data interpretation. Nucleic Acids Res. *51*, W387–W396. https://doi.org/10.1093/nar/gkad357.

70. Fang, H., and Gough, J. (2014). The 'dnet' approach promotes emerging research on cancer patient survival. Genome Med. *6*, 64. https://doi.org/10.1186/s13073-014-0064-8.

71. Rodchenkov, I., Babur, O., Luna, A., Aksoy, B.A., Wong, J.V., Fong, D., Franz, M., Siper, M.C., Cheung, M., Wrana, M., et al. (2020). Pathway Commons 2019 Update: Integration, analysis and exploration of pathway data. Nucleic Acids Res. *48*, D489–D497. https://doi.org/10.1093/nar/gkz946.

STAR★METHODS

KEY RESOURCES TABLE

REAGENT or RESOURCE	SOURCE	IDENTIFIER
Deposited data		
Cross-disease GWAS summary data for neuropsychiatric disorders	Lee et al.[18]	N/A
Cross-disease GWAS summary data for inflammatory disorders	Ellinghaus et al.[19]	N/A
GWAS Catalog	Sollis et al.[62]	https://www.ebi.ac.uk/gwas
Promoter capture Hi-C for 27 human cell and tissue types	Jung et al.[32]	N/A
Promoter capture Hi-C for 4 human neural cells	Song et al.[33]	N/A
eQTL Catalog	Kerimov et al.[34]	https://www.ebi.ac.uk/eqtl
Open Targets	Ochoa et al.[37]	https://www.opentargets.org
ChEMBL	Mendez et al.[63]	https://www.ebi.ac.uk/chembl
RCSB Protein Data Bank	Burley et al.[51]	https://www.rcsb.org
Pleiotropy-based target resources for neuropsychiatric disorders and inflammatory disorders	This study	http://www.genetictargets.com/PIPE
Software and algorithms		
fpocket	Schmidtke et al.[64]	https://github.com/Discngine/fpocket
supraHex	Fang & Gough.[46]	https://bioconductor.org/packages/supraHex
PIPE	This study	https://doi.org/10.6084/m9.figshare.25331527.v1 and https://hfang-bristol.github.io/PIPE

RESOURCE AVAILABILITY

Lead contact

Further information and request for resources should be directed to and will be fulfilled by the lead contact, Prof. Hai Fang (fh12355@rjh.com.cn).

Materials availability

This study did not generate new unique reagents.

Data and code availability

- All data used and generated in the current study are accessible through the dedicated web portal at http://www.genetictargets.com/PIPE. This portal provides an interactive platform for exploring pleiotropy-based target resources for neuropsychiatric disorders and inflammatory disorders, as well as functional modules and the prioritisation map. In support of reproducible research, we have provided comprehensive showcases that empower users to reproduce all findings from this study. These showcases include input data, line-by-line codes, and both tabular and graphical outputs, all encapsulated within a single self-contained HTML file. This file also contains detailed, step-by-step instructions on how to use the showcases and what results to expect.
- The code for PIPE is also available at https://hfang-bristol.github.io/PIPE and https://doi.org/10.6084/m9.figshare.25331527.v1.
- Any additional information required to reanalyse the data reported in this paper is available from the lead contact upon request.

METHOD DETAILS

Overview of PIPE

PIPE was implemented as an R package, specially designed for *ab initio* resolving cross-disease pleiotropic association data, as described below in the subsection 'processing input cross-disease pleiotropic association data'. Its primary objective is to accelerate

translational use in therapeutic target identification and validation. Notably, PIPE is not devised for the upstream generation of pleiotropic association data but is rather tailored to support downstream applications in therapeutic target discovery.

PIPE operates as a pleiotropy-driven unsupervised tool, featuring two integral components (*prioritisation* and *evaluation*). The resulting resources are made accessible via a web-based portal, and all showcases are made reproducible as well (Figure 1A).

The *prioritisation* component of PIPE consists of three sequential steps to accomplish therapeutic target identification at both the gene and pathway crosstalk levels, as graphically illustrated in Figure 1B.

- *Step 1.* Identify genomic/pleiotropic genes with genomic evidence by leveraging multimodal regulatory genomic datasets (see 'identification of pleiotropic genes with genomic evidence' for greater details) and networked/peripheral genes with network evidence by leveraging knowledge of protein interactions (see 'identification of peripheral genes with network evidence'), to prepare informative predictors (see 'extraction of informative predictors and preparation of gene-predictor matrix').
- *Step 2.* Combine informative predictors to prioritise target genes across the genome (that is, targets at the gene level; see 'prioritisation of targets at the gene level').
- *Step 3.* Identify a network of genes that mediate crosstalk between molecular pathways (that is, targets at the pathway crosstalk level; see 'identification of targets at the pathway crosstalk level'), followed by crosstalk-based advanced analyses, including network modular analysis (see 'crosstalk-based network modular analysis') and prioritisation map analysis (see 'crosstalk-based prioritisation map analysis').

The *evaluation* component of PIPE facilitates three instrumental analyses to enable performance evaluations, as graphically illustrated in Figure 1C.

- *Analysis 1.* Define clinical proof-of-concept targets (see 'defining clinical proof-of-concept targets') and simulate negative controls (see 'simulating negative targets as controls').
- *Analysis 2.* Do benchmarking to compare prioritisation performance in terms of separating clinical proof-of-concept targets from simulated controls (see 'benchmarking for performance comparisons').
- *Analysis 3.* Evaluate the potential of pleiotropy in informing clinical proof-of-concept therapeutics (see 'leading prioritisation analysis (LPA)' and 'pleiotropy informing clinical therapeutics (PICT)') and determine target-level disease relationships (see 'determining target-level disease relationships').

The *prioritization* component of PIPE

Processing input cross-disease pleiotropic association data

Input pleiotropic association data were a list of SNPs, each exerting pleiotropic effects on multiple diseases, along with summary statistics indicating the significance level (i.e., pleiotropic p-values combined across multiple diseases). These pleiotropic loci were identified from cross-disease GWASs (illustrated in Figure 1A). In this study, the input data encompassed a total of 142 lead SNPs (significant at p-value $<5 \times 10^{-8}$) associated with two or more neuropsychiatric disorders,[18] and a total of 224 lead SNPs (p-value $<5 \times 10^{-8}$) associated with two or more inflammatory disorders.[19] These data, manually curated in the NHGRI-EBI GWAS Catalog,[62] included pleiotropic lead SNPs and their p-values based on the European population for subsequent analysis. Additional SNPs in linkage disequilibrium (LD; $R^2 \geq 0.8$) with lead SNPs were calculated according to the European population.[65] The rationale for employing LD expansion on lead SNPs is the ease of its widespread applicability. While an alternative approach via fine-mapping could be explored in the future, it is currently constrained by the availability of summary statistics, which may not always be readily accessible. Lead and LD SNPs were scored by considering pleiotropic association p-values and LD strength R^2 (Equation 1).

$$S_{SNP} = R^2 \times \left(log_{10}\frac{1 - P_{SNP}}{P_{SNP}} - log_{10}\frac{1 - 5 \times 10^{-8}}{5 \times 10^{-8}} \right) \qquad \text{Equation 1}$$

Where S_{SNP} and P_{SNP} denote the SNP score and pleiotropic p-value combined across multiple diseases, respectively, and R^2 is the LD strength.

Identification of pleiotropic genes with genomic evidence

Lead and LD SNPs from the aforementioned pleiotropic association data were used to identify pleiotropic genes. These pleiotropic genes were categorised into three types according to the genomic evidence (i.e., which regulatory genomic datasets were used): (i) nearby genes (*nGene*), identified with evidence of genomic proximity; (ii) conformation genes (*cGene*) identified using promoter capture Hi-C datasets, reflecting evidence of gene promoters physically interacting with SNP-harbouring genomic regions; and (iii) expression-associated genes (*eGene*) identified based on eQTL datasets, reflecting evidence of genetic regulation of gene expression. Notably, immune- and brain-related datasets were used in this study, including 24 promoter capture Hi-C datasets[32,33] and 69 eQTL datasets[34]; accordingly, there were 24 sets of *cGenes* and 69 sets of *eGenes*. The scoring for *nGene* considered the influential range of distance (Equation 2). The scoring for *cGene* (or *eGene*) was tissue- or cell type-specific: for each tissue *cGene* (or *eGene*), it considered the empirical cumulative distribution function (eCDF) of the strength or significance level linking an SNP to a gene (Equations 3 and 4). The eCDF was estimated based on all SNP-gene pairs, ensuring the scaling into the 0–1 range. It is also worth noting that these three types of genes could overlap; a gene might simultaneously be categorised as both *nGene* and *eGene*.

$$S_{nGene} = \max_{SNP \in \Omega} S_{SNP} \qquad \text{Equation 2}$$

Where S_{nGene} is the score for *nGene*, S_{SNP} is the SNP score, Ω stands for collections of lead and LD SNPs located within 20kb of *nGene*, and *max* denotes the maximum scoring scheme, keeping the most informative one when multiple SNPs are located within the same pleiotropic region. In simpler terms, any genes within ±20kb around the lead and LD SNPs will be considered as *nGenes*. Notably, the findings were robust, with the trivial effect of changing the distance windows on the performance (Figure S6).

$$x = -\log_{10}(S_{SNP \leftrightarrow Gene}) \qquad \text{Equation 3}$$

$$S_{cGene} \text{ or } S_{eGene} = \max_{SNP \in \Omega} [S_{SNP} \times eCDF(x)] \qquad \text{Equation 4}$$

Where S_{cGene} is the score for *cGene*, S_{eGene} is the score for *eGene*, S_{SNP} is the SNP score, $S_{SNP \leftrightarrow Gene}$ denotes the strength or significance level linking an SNP to a gene (that is, the significance level of genetic association with gene expression for an eQTL dataset, and the physical interaction strength with a gene promoter for a promoter capture Hi-C dataset), Ω stands for collections of lead and LD SNPs, and *max* denotes the maximum scoring scheme. Notably, the scoring for *cGene* (or *eGene*) was handled for each tissue or cell type.

Identification of peripheral genes with network evidence

We employed a random walk with restart algorithm to identify peripheral genes under network influence, that is, peripheral genes connected or networked to pleiotropic genes. Each type of pleiotropic genes (or dataset of the same evidence type) was considered as core seeds, and we used them to further identify peripheral genes by leveraging the network connectivity or affinity of protein interaction information. The protein interaction data were sourced from the STRING database,[35] focusing on interactions with high-confidence scores ($\geq$700) and high-quality sources (limited to evidence codes 'experiments' or 'databases'). This corresponded to approximately 15,000 nodes or genes that underwent prioritisation.

Starting from a seed node, the random walker faces two choices at each iterative step: either moving to a randomly chosen neighbor or jumping back to the seed node (Equation 5). Upon reaching stability after a finite number of steps, the resultant steady probability vector measures the "influential impact" over the network imposed by the starting seed nodes (Equation 6). Affinity scores were computed for each gene based on its connectivity to pleiotropic genes, and the vector comprising these scores constituted a predictor for each evidence type or dataset of the same evidence type. Without a loss of generality, a gene with a higher connectivity to pleiotropic genes received a higher affinity score, with highly networked pleiotropic genes securing markedly higher affinity scores. We generated one predictor for *nGene*, 24 predictors for promoter capture Hi-C datasets (*cGenes*), and 69 predictors for eQTL datasets (*eGenes*).

$$\overrightarrow{P_t} = (1-\gamma) \times A \times \overrightarrow{P_{t-1}} + \gamma \times \overrightarrow{P_0} \qquad \text{Equation 5}$$

$$\overrightarrow{P_\infty} = \lim_{t \to \infty} \overrightarrow{P_t} \qquad \text{Equation 6}$$

Where $\overrightarrow{P_\infty}$ represents the steady probability vector (attained upon reaching stability) that stores the affinity score of all nodes in the network to the starting seed nodes (i.e., pleiotropic genes), $\overrightarrow{P_t}$ is the probability vector dictating the walker's visits to network nodes at step t, $\overrightarrow{P_0}$ is the starting probability vector that contains gene scores for seed nodes (i.e., scores in Equations 2 and 4) and 0 otherwise, γ is the restarting probability (probability of jumping back to a seed node, and $1-\gamma$ for the probability of moving to a neighbor), and A is the normalised Laplacian adjacency matrix of the network being traversed.

Extraction of informative predictors and preparation of gene-predictor matrix

As illustrated at *Step 1* of Figure 1B, we used random forest to assess the relative importance of predictors. We retained only informative predictors, identified as those whose removal resulted in a substantial decrease in accuracy; see detailed methodology.[38] Since the *nGene* predictor was considered the baseline, we retained subsets of *cGene* and *eGene* predictors that were deemed at least as important as *nGene*. In essence, utilising pleiotropic association data, we prepared a gene-predictor matrix consisting of affinity scores, where informative predictors as columns and pleiotropic and peripheral genes as rows (totaling approximately 15,000 genes).

Prioritization of targets at the gene level

We performed the gene-level target prioritisation by combining informative predictors using a method akin to Fisher's combined meta-analysis. For each predictor in the gene-predictor matrix, we converted affinity scores into *P*-like values through eCDF based on the affinity scores for all genes in that predictor (Equation 7). Then, for each gene in the gene-predictor matrix, we combined these converted p-values across predictors using Fisher's combined method (Equations 8, 9, and 10). This method is suitable when the evidence is concentrated or moderately strong.[66] Finally, we rescaled the combined p-value into a credit score, ranging from 0 to 10 (Equation 11).

$$P_i^j = eCDF\left(AF_i^j\right) \qquad \text{Equation 7}$$

CellPress
OPEN ACCESS

Where AF_i^j denotes the affinity score for the ith gene in terms of the jth predictor, P_i^j is the corresponding converted p-value, and *eCDF* is estimated based on all genes.

$$x_i = -2\sum_{j}^{J} \log P_i^j \quad \text{Equation 8}$$

$$x_i \sim \chi^2(2J) \quad \text{Equation 9}$$

$$CP_i = CDF(x_i) \quad \text{Equation 10}$$

$$CS_i = 10 \times \frac{-\log CP_i - MIN_k^K(-\log CP_k)}{MAX_k^K(-\log CP_k) - MIN_k^K(-\log CP_k)} \quad \text{Equation 11}$$

Where P_i^j denotes the converted p-value for the ith gene in terms of the jth predictor, J is the number of the informative predictors, $\chi^2(2J)$ denotes the Chi-squared distribution with $2J$ degrees of freedom, CP_i represents the combined p-value for the ith gene (i.e., eCDF of the Chi-squared distribution valued at x_i), and CS_i is the credit score for the ith gene (among K genes).

Identification of targets at the pathway crosstalk level

First, we constructed a gene network by merging KEGG pathways.[67] Next, we extended our previously established algorithm[68–70] to identify a subset of the pathway-merged gene network that contains highly prioritised/credited and interconnected genes. This identified gene subnetwork, referred to as 'pathway crosstalk', underwent a degree-preserving node permutation test to estimate its significance (p-value) relative to chance occurrences (i.e., by counting how frequently it would occur by chance; see detailed information[70]).

The *evaluation* component of PIPE

Defining clinical proof-of-concept targets

Information on current therapeutics, including drugs, development phases, target genes, and disease indications, was retrieved from the ChEMBL database.[63] Specifically, for a particular disease, we defined '*clinical proof-of-concept targets*' as therapeutic genes targeted by drugs that have advanced to development phase 2 or above, signifying substantial evidence of their efficacy in treating the respective disease.

Simulating negative targets as controls

In our performance evaluation, negative targets were simulated for a particular disease following the strategy outlined in *Analysis 1* of Figure 1C, with two key steps. The first step is to define druggable landscape. We delineated the druggable landscape, encompassing all targetable genes across various disease indications and at all drug development phases. The second step is to simulate negative targets. These negative targets were simulated by selecting genes from the druggable landscape, but after systematically excluding genes known to be specific to the disease under consideration, along with their interacting neighbors. Interacting neighbors were determined based on interaction data sourced from databases including STRING[35] and Pathway Commons.[71] Taken together, this simulation strategy aimed to ensure that the negative targets, under the constraint of the existing druggable landscape (i.e., evidence already explored) and protein interaction information (due to the tendency of known targets to interact together), were unlikely to be target genes for the disease under consideration.

Benchmarking for performance comparisons

A benchmarking analysis was conducted to assess and compare the performance of PIPE with the state-of-the-art Open Targets.[37] The performance focused on the ability to distinguish clinical proof-of-concept drug targets from simulated negative targets. In the Open Targets model, the prioritisations were evaluated based on individual pieces of evidence, specifically *genetic association* (for details see the open targets genetics portal[36]) and *text mining*. Information on *genetic association* scores and *text mining* scores was extracted from the Open Targets Platform, accessible at https://platform.opentargets.org.[37]

Leading prioritization analysis (LPA)

LPA was performed to quantify the extent of enrichment in a target set, such as clinical proof-of-concept targets, at the 'leading prioritisation' within the prioritised gene list ranked by credit scores. Analogous to gene set enrichment analysis, the leading prioritisation (or the leading edge) encompasses the leftmost subset of the highly prioritised/credited gene list. The normalised enrichment score (NES), calculated as the observed running enrichment score divided by the expected score, was determined, with the significance level (p-value) estimated through a permutation test conducted 50,000 times.

Pleiotropy informing clinical therapeutics (PICT)

We introduced a new metric, PICT, to quantify the Pleiotropy Informing Clinical Therapeutics potential for a particular disease. PICT measures the inclination of the prioritised gene list to contain clinical proof-of-concept targets. This tendency was assessed through

LPA, evaluating the degree to which clinical proof-of-concept targets are enriched at the top/leftmost of the prioritised gene list. PICT was computed using the formula specified in Equation 12, where diseases with higher PICT exhibit more substantial enrichment (FDR; p-value adjusted accounting for multiple tests), a higher NES, and a higher coverage (Coverage, defined as the fraction of clinical proof-of-concept targets recovered at the 'leading prioritisation' of the prioritised list).

$$PICT = \log_{10}(NES \times Coverage \; / \; FDR) \qquad \text{Equation 12}$$

Determining target-level disease relationships

We presented the relationships among diseases as a network, with diseases represented as nodes and their estimated relations as edges. For each disease, member genes were defined as clinical proof-of-concept targets recovered at the leading prioritisation. We initially inferred edges if any member genes were shared between diseases and subsequently refined by identifying a minimum spanning tree. Only edges present in the resulting tree were retained. The thickness of an edge was proportional to the number of member genes shared between the two endpoint diseases.

QUANTIFICATION AND STATISTICAL ANALYSIS

Crosstalk-based network modular analysis

We employed a spin-glass model and simulated annealing to identify network modules within crosstalks. For each module, we performed enrichment analysis to unveil its functional relevance, therapeutic potential, and evolutionary origin. Functional relevance was determined by identifying enriched KEGG pathways. We identified enriched approved drug targets and non-approved phased drug targets using the ChEMBL database[63] to assess therapeutic potential. Therapeutic targets were categorised into two groups: one for approved drug targets (i.e., genes targeted by any approved drugs), and the other for non-approved phased drug targets (i.e., genes targeted by non-approved phased drugs). Additionally, genes created in each of 16 phylostrata, arranged from the earliest (PSG01) to the most recent (PSG16), were obtained from this study[44] for enrichment analysis to reveal evolutionary origin.

Crosstalk-based prioritisation map analysis

We identified two instances of pathway crosstalks, one associated with neuropsychiatric disorders and the other with inflammatory disorders, yielding a total of 116 crosstalk target genes. We utilised the supraHex package[46] to cluster and visualise these genes on a supra-hexagonal map consisting of 91 hexagons. The map was trained using a matrix containing credit scores for the 116 target genes across two systems of disorders (i.e., neuropsychiatric and inflammatory). This trained map was used to illustrate a gene prioritisation profile for each disorder system. The trained map was also topologically divided into target gene clusters. We overlaid the trained map with druggable pocket data, estimating the probability of each hexagon containing structurally targetable genes. A gene was considered targetable if predicted to harbor drug-like binding sites (i.e., druggable pockets) based on its known protein structure(s). All known protein structures were sourced from the PDB database,[51] with druggable pockets predicted using the fpocket software.[64]

Cell Reports Methods, Volume *4*

Supplemental information

A cross-disease, pleiotropy-driven approach for therapeutic target prioritization and evaluation

Chaohui Bao, Tingting Tan, Shan Wang, Chenxu Gao, Chang Lu, Siyue Yang, Yizhu Diao, Lulu Jiang, Duohui Jing, Liye Chen, Haitao Lv, and Hai Fang

Supplemental Figures

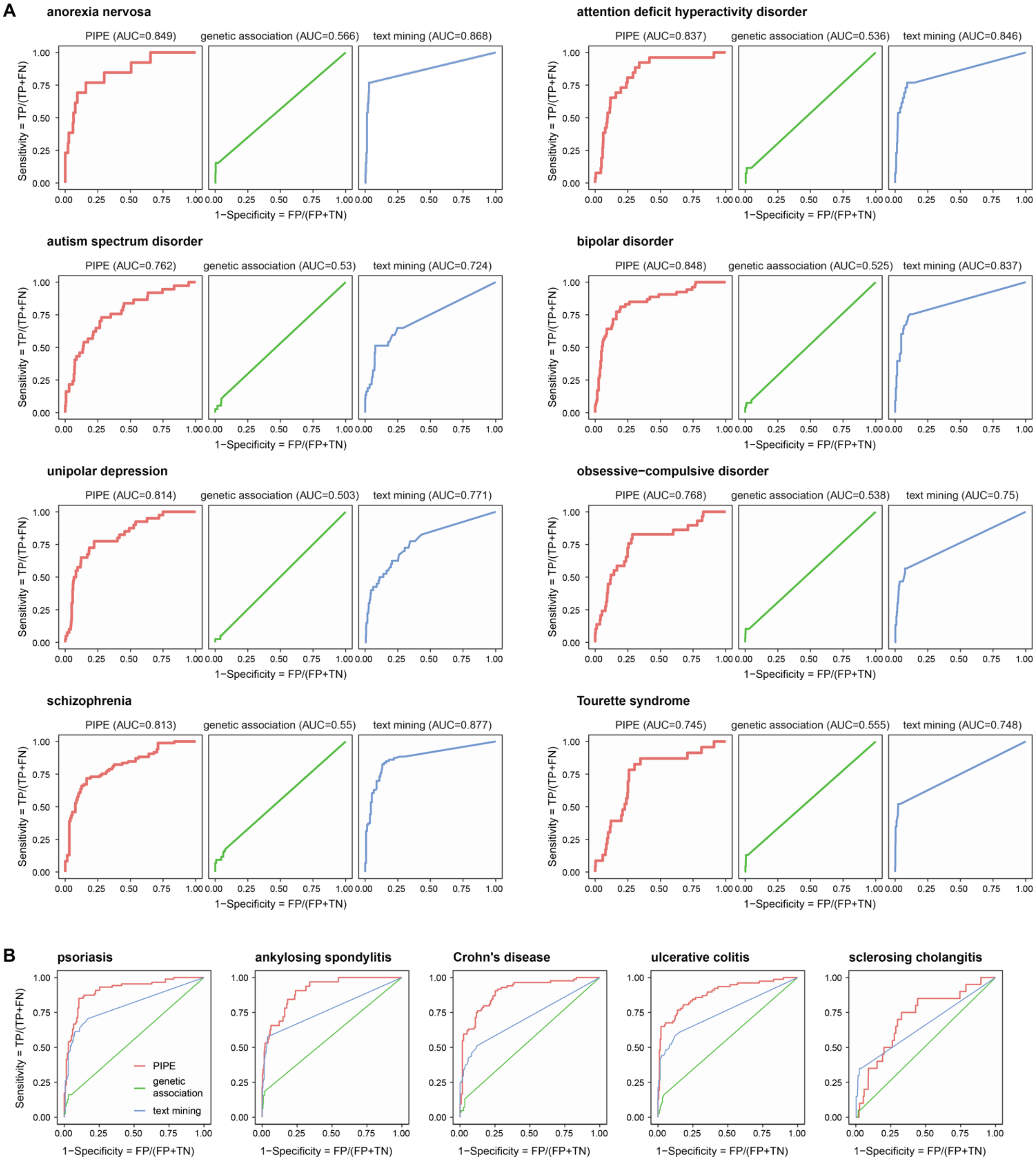

Figure S1. AUC plots comparing the performance between PIPE and Open Targets (including 'genetic association' and 'text mining'), related to Figure 2A and Figure 4A. FN, false negatives; FP, false positives; TN, true negatives; TP: true positives. **(A)** AUC plots for each individual disease within neuropsychiatric disorders. **(B)** AUC plots for each individual disease within inflammatory disorders.

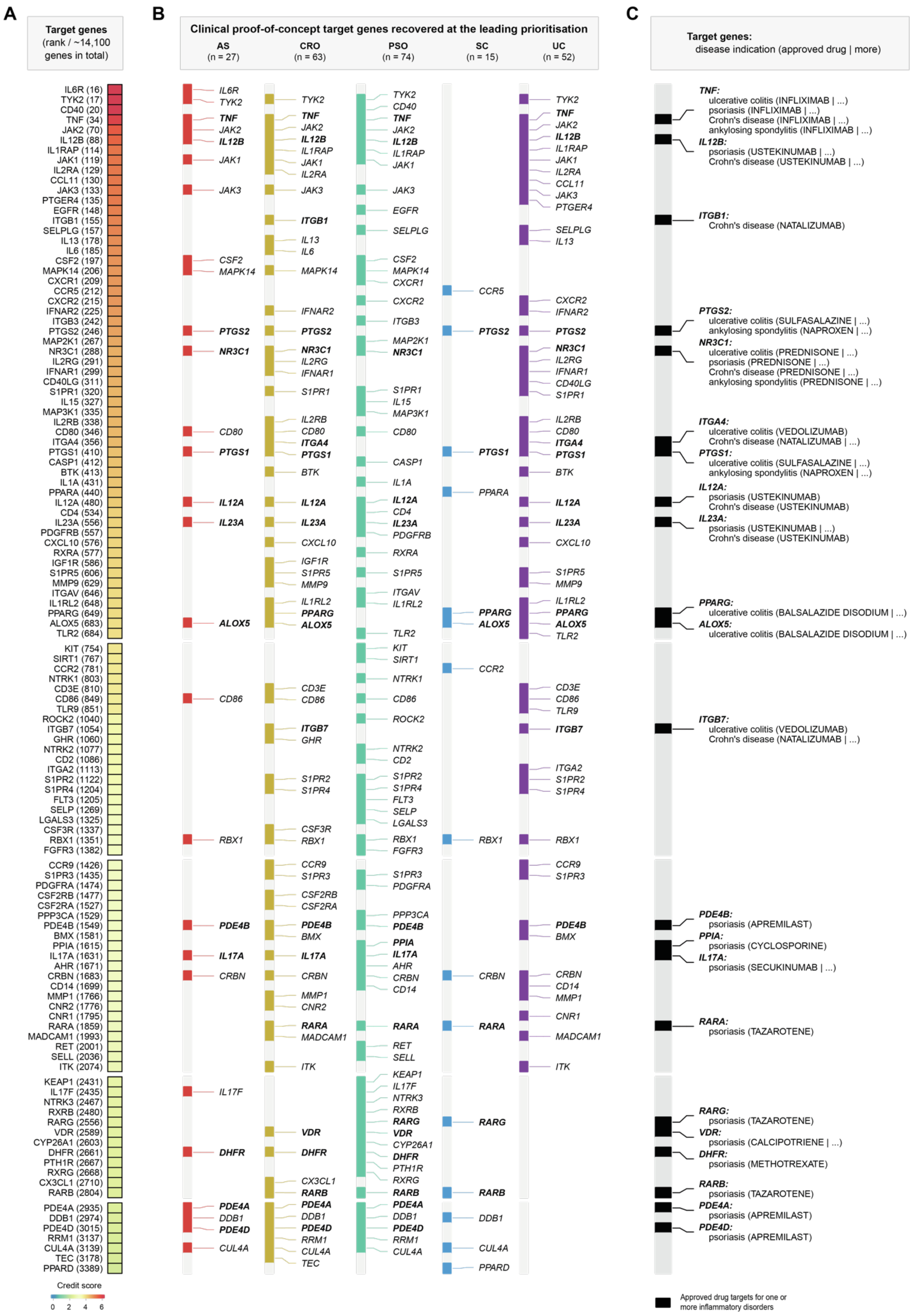
A
Target genes (rank / ~14,100 genes in total)
IL6R (16)
TYK2 (17)
CD40 (20)
TNF (34)
JAK2 (70)
IL12B (88)
IL1RAP (114)
JAK1 (119)
IL2RA (129)
CCL11 (130)
JAK3 (133)
PTGER4 (135)
EGFR (148)
ITGB1 (155)
SELPLG (157)
IL13 (178)
IL6 (185)
CSF2 (197)
MAPK14 (206)
CXCR1 (209)
CCR5 (212)
CXCR2 (215)
IFNAR2 (225)
ITGB3 (242)
PTGS2 (246)
MAP2K1 (267)
NR3C1 (288)
IL2RG (291)
IFNAR1 (299)
CD40LG (311)
S1PR1 (320)
IL15 (327)
MAP3K1 (335)
IL2RB (338)
CD80 (346)
ITGA4 (356)
PTGS1 (410)
CASP1 (412)
BTK (413)
IL1A (431)
PPARA (440)
IL12A (480)
CD4 (534)
IL23A (556)
PDGFRB (557)
CXCL10 (576)
RXRA (577)
IGF1R (586)
S1PR5 (606)
MMP9 (629)
ITGAV (646)
IL1RL2 (648)
PPARG (649)
ALOX5 (683)
TLR2 (684)
KIT (754)
SIRT1 (767)
CCR2 (781)
NTRK1 (803)
CD3E (810)
CD86 (849)
TLR9 (851)
ROCK2 (1040)
ITGB7 (1054)
GHR (1060)
NTRK2 (1077)
CD2 (1086)
ITGA2 (1113)
S1PR2 (1122)
S1PR4 (1204)
FLT3 (1205)
SELP (1269)
LGALS3 (1325)
CSF3R (1337)
RBX1 (1351)
FGFR3 (1382)
CCR9 (1426)
S1PR3 (1435)
PDGFRA (1474)
CSF2RB (1477)
CSF2RA (1527)
PPP3CA (1529)
PDE4B (1549)
BMX (1581)
PPIA (1615)
IL17A (1631)
AHR (1671)
CRBN (1683)
CD14 (1699)
MMP1 (1766)
CNR2 (1776)
CNR1 (1795)
RARA (1859)
MADCAM1 (1993)
RET (2001)
SELL (2036)
ITK (2074)
KEAP1 (2431)
IL17F (2435)
NTRK3 (2467)
RXRB (2480)
RARG (2556)
VDR (2589)
CYP26A1 (2603)
DHFR (2661)
PTH1R (2667)
RXRG (2668)
CX3CL1 (2710)
RARB (2804)
PDE4A (2935)
DDB1 (2974)
PDE4D (3015)
RRM1 (3137)
CUL4A (3139)
TEC (3178)
PPARD (3389)
Credit score
0 2 4 6
B
Clinical proof-of-concept target genes recovered at the leading prioritisation
AS (n = 27)
CRO (n = 63)
PSO (n = 74)
SC (n = 15)
UC (n = 52)
C
Target genes:
disease indication (approved drug | more)
TNF:
ulcerative colitis (INFLIXIMAB | ...)
psoriasis (INFLIXIMAB | ...)
Crohn's disease (INFLIXIMAB | ...)
ankylosing spondylitis (INFLIXIMAB | ...)
IL12B:
psoriasis (USTEKINUMAB | ...)
Crohn's disease (USTEKINUMAB | ...)
ITGB1:
Crohn's disease (NATALIZUMAB)
PTGS2:
ulcerative colitis (SULFASALAZINE | ...)
ankylosing spondylitis (NAPROXEN | ...)
NR3C1:
ulcerative colitis (PREDNISONE | ...)
psoriasis (PREDNISONE | ...)
Crohn's disease (PREDNISONE | ...)
ankylosing spondylitis (PREDNISONE | ...)
ITGA4:
ulcerative colitis (VEDOLIZUMAB)
Crohn's disease (NATALIZUMAB | ...)
PTGS1:
ulcerative colitis (SULFASALAZINE | ...)
ankylosing spondylitis (NAPROXEN | ...)
IL12A:
psoriasis (USTEKINUMAB)
Crohn's disease (USTEKINUMAB)
IL23A:
psoriasis (USTEKINUMAB | ...)
Crohn's disease (USTEKINUMAB)
PPARG:
ulcerative colitis (BALSALAZIDE DISODIUM | ...)
ALOX5:
ulcerative colitis (BALSALAZIDE DISODIUM | ...)
ITGB7:
ulcerative colitis (VEDOLIZUMAB)
Crohn's disease (NATALIZUMAB | ...)
PDE4B:
psoriasis (APREMILAST)
PPIA:
psoriasis (CYCLOSPORINE)
IL17A:
psoriasis (SECUKINUMAB | ...)
RARA:
psoriasis (TAZAROTENE)
RARG:
psoriasis (TAZAROTENE)
VDR:
psoriasis (CALCIPOTRIENE | ...)
DHFR:
psoriasis (METHOTREXATE)
RARB:
psoriasis (TAZAROTENE)
PDE4A:
psoriasis (APREMILAST)
PDE4D:
psoriasis (APREMILAST)
Approved drug targets for one or more inflammatory disorders

Figure S2. Illustration of clinical proof-of-concept therapeutic targets recovered at the leading prioritisation for each individual disease within inflammatory disorders, related to Figure 4. (A) The heatmap for clinical proof-of-concept therapeutic targets color-coded by the credit score. The number in the parentheses indicates the target rank. **(B)** Annotations to clinical proof-of-concept targets per disease as indicated. **(C)** Annotations for approved drug targets, disease indications, and approved drugs. This illustration implicates drug repurposing opportunities within inflammatory disorders.

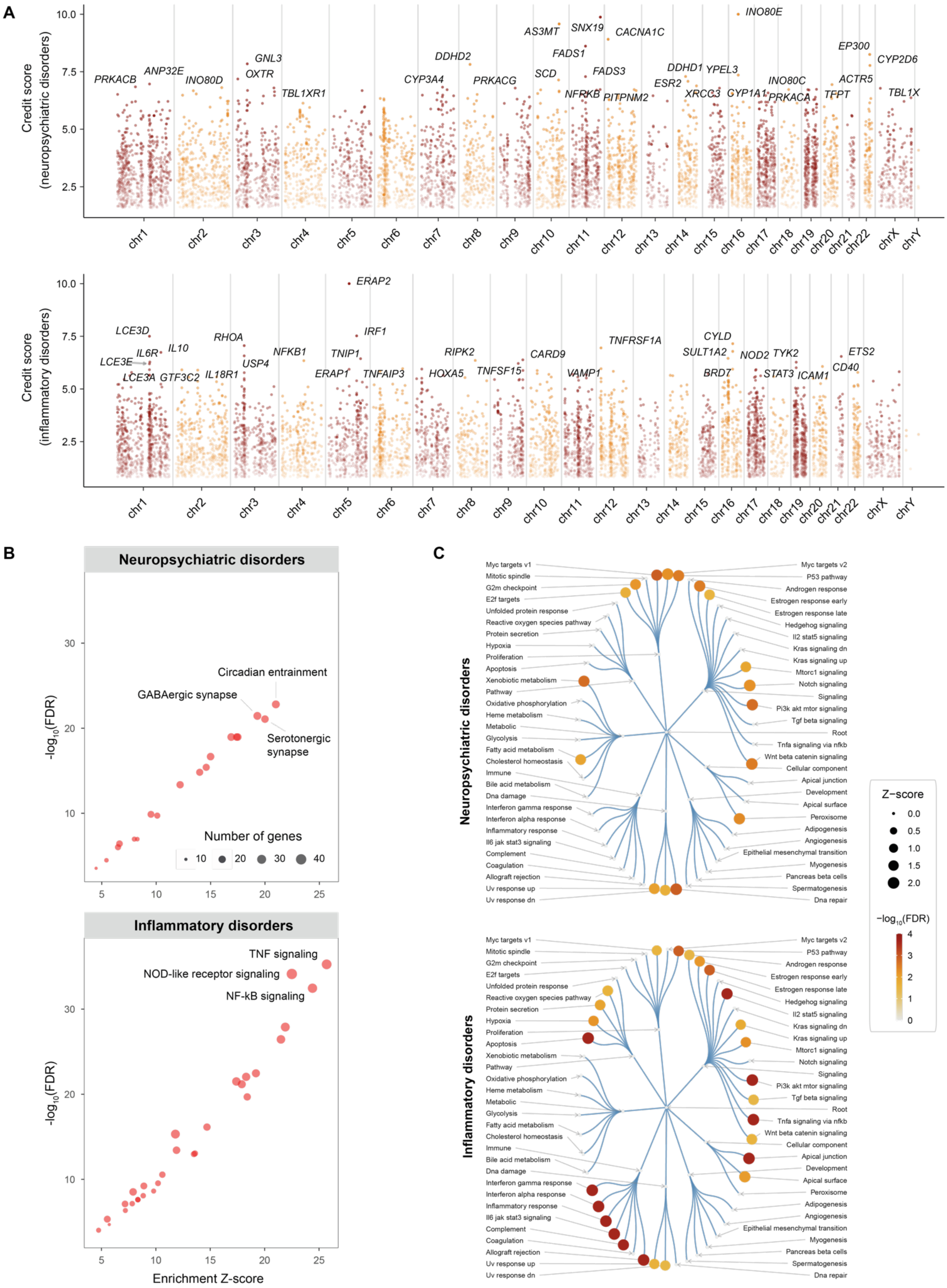
A
Credit score (neuropsychiatric disorders)
10.0
7.5
5.0
2.5
PRKACB
ANP32E
INO80D
GNL3
OXTR
TBL1XR1
DDHD2
CYP3A4
PRKACG
AS3MT
SNX19
CACNA1C
FADS1
SCD
FADS3
NFRKB
PITPNM2
DDHD1
YPEL3
ESR2
XRCC3
CYP1A1
INO80E
INO80C
PRKACA
EP300
CYP2D6
ACTR5
TFPT
TBL1X
chr1
chr2
chr3
chr4
chr5
chr6
chr7
chr8
chr9
chr10
chr11
chr12
chr13
chr14
chr15
chr16
chr17
chr18
chr19
chr20
chr21
chr22
chrX
chrY
Credit score (inflammatory disorders)
ERAP2
LCE3D
IL6R
IL10
LCE3E
LCE3A
GTF3C2
RHOA
USP4
IL18R1
NFKB1
TNIP1
IRF1
ERAP1
TNFAIP3
RIPK2
HOXA5
TNFSF15
CARD9
VAMP1
TNFRSF1A
CYLD
SULT1A2
NOD2
TYK2
BRD7
STAT3
ICAM1
ETS2
CD40
B
Neuropsychiatric disorders
Circadian entrainment
GABAergic synapse
Serotonergic synapse
Number of genes
10
20
30
40
-log10(FDR)
Inflammatory disorders
TNF signaling
NOD-like receptor signaling
NF-kB signaling
Enrichment Z-score
C
Neuropsychiatric disorders
Inflammatory disorders
Myc targets v1
Mitotic spindle
G2m checkpoint
E2f targets
Unfolded protein response
Reactive oxygen species pathway
Protein secretion
Hypoxia
Proliferation
Apoptosis
Xenobiotic metabolism
Pathway
Oxidative phosphorylation
Heme metabolism
Metabolic
Glycolysis
Fatty acid metabolism
Cholesterol homeostasis
Immune
Bile acid metabolism
Dna damage
Interferon gamma response
Interferon alpha response
Inflammatory response
Il6 jak stat3 signaling
Complement
Coagulation
Allograft rejection
Uv response up
Uv response dn
Myc targets v2
P53 pathway
Androgen response
Estrogen response early
Estrogen response late
Hedgehog signaling
Il2 stat5 signaling
Kras signaling dn
Kras signaling up
Mtorc1 signaling
Notch signaling
Signaling
Pi3k akt mtor signaling
Tgf beta signaling
Root
Tnfa signaling via nfkb
Wnt beta catenin signaling
Cellular component
Apical junction
Development
Apical surface
Peroxisome
Adipogenesis
Angiogenesis
Epithelial mesenchymal transition
Myogenesis
Pancreas beta cells
Spermatogenesis
Dna repair
Z-score
0.0
0.5
1.0
1.5
2.0
-log10(FDR)
4
3
2
1
0

Figure S3. Target genes prioritisations, pathway enrichments, and hallmark enrichments, related to Figure 5. (A) Manhattan plots illustrating credit scores (y-axis) for all prioritised target genes across chromosomes (x-axis), with top 30 named. *Top panel:* neuropsychiatric disorders. *Bottom panel:* inflammatory disorders. **(B)** Dot plots illustrating top KEGG pathways enriched for each system of disorders, with the top 3 named. *Left panel:* neuropsychiatric disorders. *Right panel:* inflammatory disorders. **(C)** Circular illustration of molecular hallmark enrichments for each system of disorders. *Left panel:* neuropsychiatric disorders. *Right panel:* inflammatory disorders.

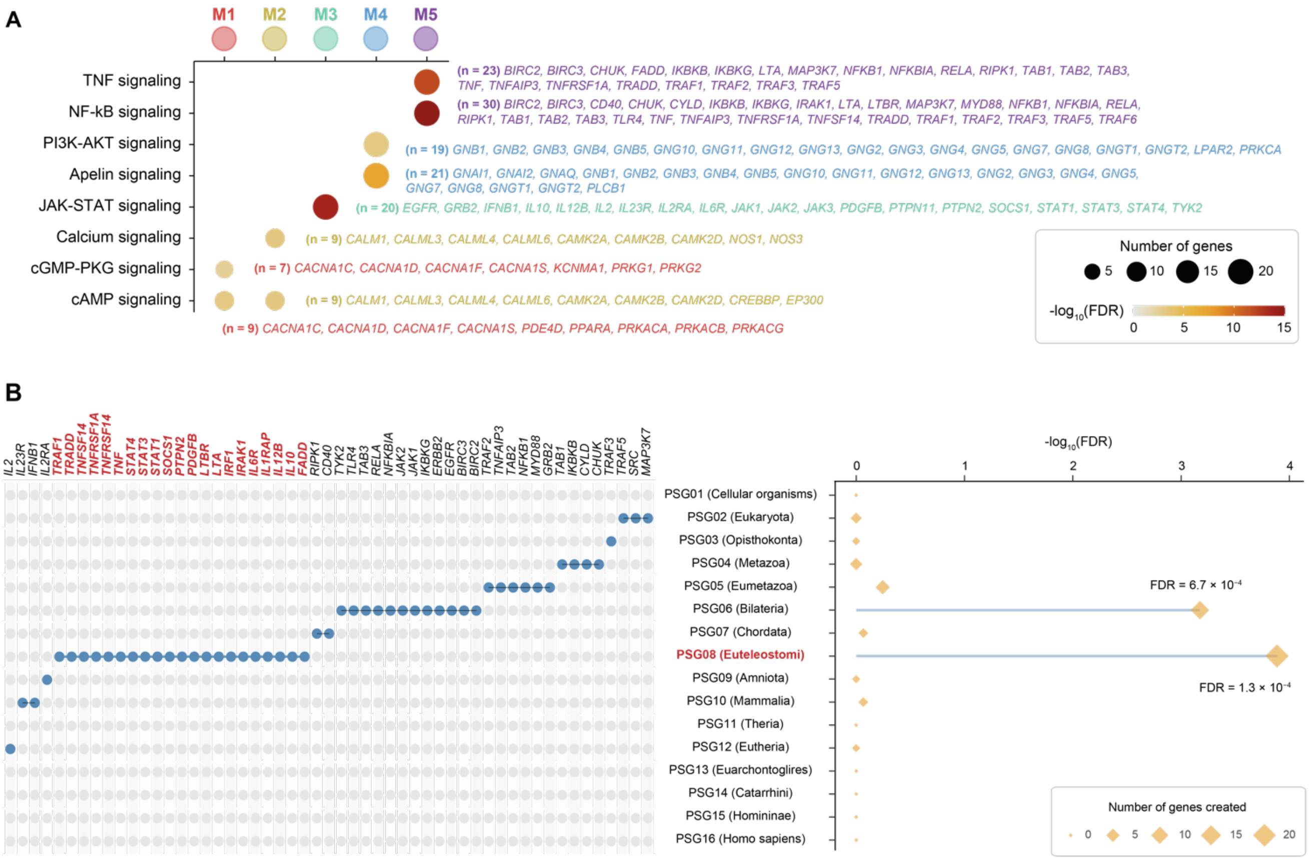

Figure S4. Crosstalk-based network modular analysis, related to Figure 6. (A) KEGG pathway enrichment analysis for genes within each of five modules (M1 – M5). Enrichment analysis was based on one-sided Fisher's exact test. **(B)** Enrichment analysis using phylostrata reveals evolutionary origins of genes in modules specific to inflammatory modules. Kite plot of enriched phylostrata (right panel), with created genes per phylostratum as indicated in blue dots (left panel). Dated through genomic phylostratigraphy (PSG), phylostrata are coded from the earliest (PSG01) to the most recent (PSG16). A phylostratum contains a group of genes that were first created at this phylostratum; for example, genes in red were first created at Euteleostomi, mostly involved in NF-kB signaling (*IRAK1*, *LTA*, *LTBR*, *TNF*, *TNFRSF1A*, *TNFSF14*, *TRADD*, and *TRAF1*) and JAK-STAT signaling (*IL10*, *IL12B*, *IL6R*, *PDGFB*, *PTPN2*, *SOCS1*, *STAT1*, *STAT3*, and *STAT4*).

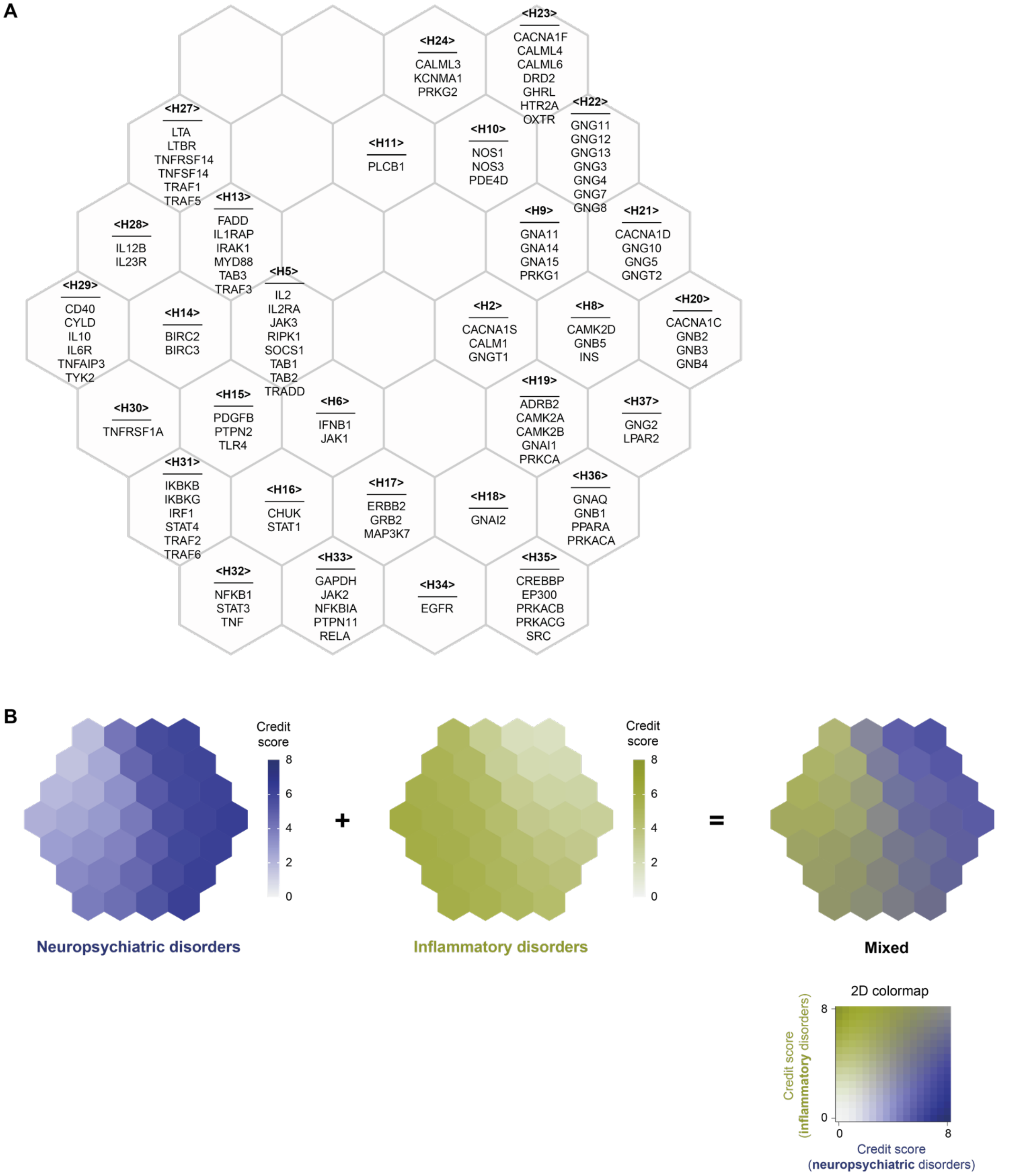

Figure S5. Crosstalk-based prioritisation map analysis, related to Figure 7. (A) A 2D map with target genes listed per hexagon (H1 - H37). **(B)** Prioritisation map coded using two mixed colors.

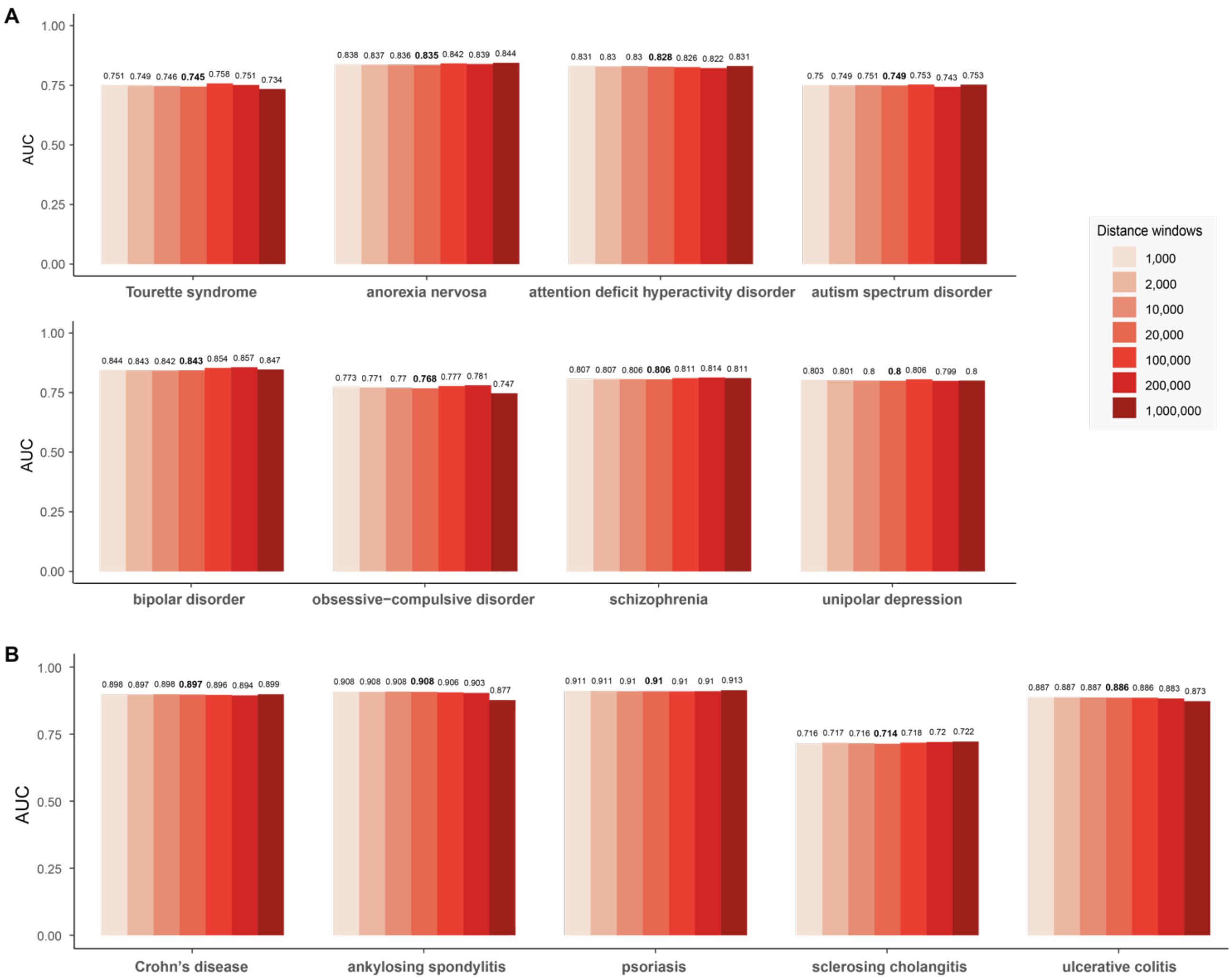

Figure S6. Performance evaluation by changing the distance windows, related to the STAR Methods. (A) Evaluation for neuropsychiatric disorders. **(B)** Evaluation for inflammatory disorders. The texts in bold highlight the selection of a 20kb distance.
